# Classification images for aerial images capture visual expertise for binocular disparity and a prior for lighting from above

**DOI:** 10.1167/jov.24.4.11

**Published:** 2024-04-12

**Authors:** Emil Skog, Timothy S. Meese, Isabel M. J. Sargent, Andrew Ormerod, Andrew J. Schofield

**Affiliations:** 1 School of Psychology, College of Health and Life Sciences, Aston University, Birmingham, B4 7ET, UK; 2 Aston Laboratory for Immersive Virtual Environments, College of Health and Life Sciences, Aston University, Birmingham, B4 7ET, UK; 3 Department of Health, Learning and Technology, Luleå University of Technology, Luleå, Sweden; 4 Ordnance Survey, Adanac Drive, Southampton, SO16 0AS, UK; 5 Electronics and Computer Science, University of Southampton, University Road, Southampton, SO17 1BJ, UK

**Keywords:** classification images, visual expertise, remote sensing, binocular disparity, lighting prior, photogrammetry, reverse correlation, aerial images

## Abstract

Using a novel approach to classification images (CIs), we investigated the visual expertise of surveyors for luminance and binocular disparity cues simultaneously after screening for stereoacuity. Stereoscopic aerial images of hedges and ditches were classified in 10,000 trials by six trained remote sensing surveyors and six novices. Images were heavily masked with luminance and disparity noise simultaneously. Hedge and ditch images had reversed disparity on around half the trials meaning hedges became ditch-like and vice versa. The hedge and ditch images were also flipped vertically on around half the trials, changing the direction of the light source and completing a 2 × 2 × 2 stimulus design. CIs were generated by accumulating the noise textures associated with “hedge” and “ditch” classifications, respectively, and subtracting one from the other. Typical CIs had a central peak with one or two negative side-lobes. We found clear differences in the amplitudes and shapes of perceptual templates across groups and noise-type, with experts prioritizing binocular disparity and using this more effectively. Contrariwise, novices used luminance cues more than experts meaning that task motivation alone could not explain group differences. Asymmetries in the luminance CIs revealed individual differences for lighting interpretation, with experts less prone to assume lighting from above, consistent with their training on aerial images of UK scenes lit by a southerly sun. Our results show that (i) dual noise in images can be used to produce simultaneous CI pairs, (ii) expertise for disparity cues does not depend on stereoacuity, (iii) CIs reveal the visual strategies developed by experts, (iv) top-down perceptual biases can be overcome with long-term learning effects, and (v) CIs have practical potential for directing visual training.

## Introduction

### General background

The creation of authoritative geographic mapping data requires careful measurement and categorization of features in the landscape. At the Ordnance Survey (OS; UK), landscape features are typically measured and categorized by experienced remote sensing surveyors based on high resolution stereoscopic photographs of aerial-view landscape imagery (photogrammetry). These photographs are taken from an aircraft, and stereogram pairs are created by using two images covering overlapping landscape areas spaced out along the flight path. The remote sensing surveyors use this stereoscopic imagery to create and update map data, including measures such as ground height, and geographic features such as fences, paths, vegetation, houses, embankments, roads, and rivers. This perceptual task is often difficult to perform accurately and requires training and experience. While it is relatively easy to teach the semantic properties of geographic features, it is more challenging to formally teach the perceptual cues required for detection and discrimination. Visual expertise is accumulated over time in the workplace, allowing experienced surveyors to better detect and classify features in images. However, even experienced surveyors find it hard to articulate the nature of their expertise, and there is little knowledge about such expertise in the literature.

Previous studies of visual expertise include work on medical imagery (e.g., Fox & Faulkner-Jones, 2017; Gegenfurtner, Lehtinen, & Säljö, 2011; Krupinski, 2010; Krupinski et al., 2006; Reingold & Sheridan, 2012; Wolfe, Evans, Drew, Aizenman, & Josephs, 2016), pilot perception in the aviation cockpit (e.g., Bellenkes, Wickens, & Kramer, 1997; Kasarskis, Stehwien, Hickox, Aretz, & Wickens, 2001; Schriver, Morrow, Wickens, & Talleur, 2008; Ziv, 2016) and 2D aerial imagery analysis (Lansdale, Underwood, & Davies, 2010; Šikl, Svatoňová, Děchtěrenko, & Urbánek, 2019). Performance metrics tend to show that experts require less time, have higher accuracy and produce fewer errors than novices in detection and classification tasks within their domain of expertise. Much of the existing literature on visual expertise has used eye-tracking to study saccades, gaze dwell time, and the number of fixations. Such studies measure how observers foveate the image, providing insight into how image properties and features prompt attentional deployment. In general, experts tend to spend less time at each fixation point before making decisions or moving on to a new fixation point. Experts also tend to make fewer fixations on task-irrelevant items and locations. Studies have also shown that experts gain more information from briefly presented (<250 ms) domain-related images (e.g., Drew, Evans, Võ, Jacobson, & Wolfe, 2013; Evans, Georgian-Smith, Tambouret, Birdwell, & Wolfe, 2013; Kundel & Nodine, 1975), suggesting that experts process more task-relevant visual information from global scene structure in the first glance. All this suggests that experts are more efficient than novices in processing global scene structure for guiding initial eye movements toward task-relevant areas of an image. Importantly, expertise is typically domain specific, and experts are no better than novices at tasks outside of their domain of expertise (Nodine & Krupinski, 1998; Reingold, Charness, Pomplun, & Stampe, 2001).

Although the research above has revealed domain-specific changes in gist processing and search strategies, eye-movement studies can say little or nothing about the information being extracted in each fixation, nor how this differs across experts and novices. Classification images (CIs) offer a possible way to do this.

The CI method is a psychophysical technique that allows researchers to characterize internal perceptual templates, illustrating visual information sampling strategies (Ahumada, 1996; Eckstein & Ahumada, 2002; Murray, 2011). In a typical CI study, observers are tasked to detect or discriminate a target that is masked by random visual noise textures. These random textures act as a generalized mask, making detection or discrimination more difficult. However, they also have a modulating effect on the target, promoting or demoting detection on a trail-by-trial basis. For example, detection of a white target would be promoted by white pixels in the noise and demoted by black pixels. To generate a CI, noise textures are accumulated from each trial depending on the observer's responses. If enough noise samples are averaged, features that support detection or discrimination of the target are revealed.

CIs have provided valuable insights into visual information sampling strategies for extracting relevant image features (Abbey & Eckstein, 2002; Abbey & Eckstein, 2006; Ahumada, 1996; Beard & Ahumada, 1998; Beard & Ahumada, 1999), illusory shapes and perceptual completion (Gold, Murray, Bennett, & Sekuler, 2000; Gosselin & Schyns, 2003; Kontsevich & Tyler, 2004), stereoscopic surfaces (Gosselin, Bacon, & Mamassian, 2004; Neri, Parker, & Blakemore, 1999), localization tasks (Abbey & Eckstein, 2014; Abbey, Lago, & Eckstein, 2021), and the spatial extent of luminance contrast pooling (Baker & Meese, 2014). The method has also been used to investigate perceptual learning (Dobres & Seitz, 2010; Gold, Sekuler, & Bennett, 2004; Kurki & Eckstein, 2014; Li, Levi, & Klein, 2004). In these studies, the perceptual templates increased in amplitude and area with practice, making greater use of relevant stimulus information. Since the ideal template is a perfect match to the target (Abbey & Eckstein, 2006; Ahumada, 1996; Beard & Ahumada, 1998; Chauvin, Worsley, Schyns, Arguin, & Gosselin, 2005), the degree of match between CIs and the target can serve as a metric for the observer's performance. Thus, CIs could be used to compare experts and novices for image-based tasks in various domains of expertise.

### The current study and specific background

Here, we test the ideas above for a set of specific predictions (described below) using the CI technique to compare the visual strategies of expert remote sensing surveyors with those of novices. We did this for the discrimination of two landscape features that have similar visual textures but dissimilar 3D relief: hedges and ditches. In principle, these can be discriminated from luminance and/or binocular disparity cues. (We consider the details of these depth cues later).

The experimental approach we developed for investigating the above is novel. By imposing spatial noise made from luminance textures and random binocular disparities onto stereoscopic landscape images we were able to derive simultaneous pairs of CIs for each observer. By examining and quantifying these we then established how observers used disparity and luminance cues when performing hedge/ditch classifications. Our image treatments involved a 2 × 2 manipulation where we flipped: (1) the disparity of half the images (to produce pseudoscopic viewing), so that hedges had ditch-like disparity profiles and vice-versa, and (2) the orientation of half the images (mirror-reversed around a horizontal axis) to change the lighting and shading cues (see below). We predicted that experts would make more use of disparity cues than novices, and thus have more clearly defined disparity CIs for two reasons. First, an informal preliminary report from author A.O.—a remote sensing instructor at the OS—, advised that hedges and ditches are typically identified according to their perceived stereoscopic relief (i.e., their 3D quality). Second, the expert surveyors in our study were more experienced than novices in making photogrammetric judgments involving disparity cues. Even so, we also expected participants to combine disparity and luminance cues instead of completely ignoring one of them because cue combination tends to support stronger stereoscopic perception (Doorschot, Kappers, & Koenderink, 2001; Hartle, Irving, Allison, Glaholt, & Wilcox, 2022; Hibbard, Goutcher, Hornsey, Hunter, & Scarfe, 2023; Lovell, Bloj, & Harris, 2012; but see Chen & Tyler, 2015 where luminance cues made disparity cues redundant).

Regarding the form of the CI templates, we expected that crossed and uncrossed disparities would promote “hedge” and “ditch” responses, respectively, regardless of the ground truth of the image owing to the unambiguous 3D relief of tall hedges and deep ditches in the real world. We also predicted that luminance would be an influential factor because luminance contrast is important for depth perception (Egusa, 1983; O'Shea, Blackburn, & Ono, 1994) under two different assumptions about shape from shading. On the assumption of diffuse lighting, surface peaks and troughs align with light and dark image regions, leading to the perception that “dark is deep” (Cooper & Norcia, 2014; Hibbard et al., 2023; Langer & Bülthoff, 2000; Langer & Zucker, 1994; Potetz & Lee, 2003; Schofield, Rock, & Georgeson, 2011; Sun & Schofield, 2012). On the assumption of punctate lighting, a single-point light source means luminance peaks are perceived as surfaces facing that light source, such as a hedge with a highlight on the side facing the sun (Adams, Graf, & Ernst, 2004; Berbaum, Bever, & Chung, 1983; Brewster, 1826; Koenderink, van Doorn, Kappers, te Pas, & Pont, 2003; Mamassian & Goutcher, 2001; Pont, van Doorn, & Koenderink, 2017; Ramachandran, 1988; Rittenhouse, 1786; Schofield, Rock, & Georgeson, 2011; Stone, Kerrigan, & Porrill, 2009; Sun & Perona, 1998; Sun & Schofield, 2012). These assumptions invoke subtly different relationships between luminance and shape. In our experiment, a diffuse lighting prior predicts a strategy of “hedges are light and ditches are dark” (dark is deep), with luminance peaks (hedges) and troughs (ditches) aligned with the center of the landscape feature. On the other hand, if the lighting is assumed to be punctate, then this predicts luminance peaks on surface slopes that face toward the assumed light source and thus an offset between such peaks and the center of the landscape feature in the direction of the assumed light source. For example, consider an observer who assumes lighting from above, by which we mean from the top of the 2D image plane (note that to avoid confusion of terminology with top/bottom and above/below in 3D, we will refer to this direction as “north,” meaning the top of the page regardless of what a compass would say). This observer would expect convex hedges to have a highlight toward the “northern” part of the feature, with less luminance in the “southern” part, representing a shadow or internal shading. Similarly, our hypothetical observer would expect a concave ditch lit from the “north” to be lighter toward the “south” of the feature, as light would not reach the “northern” concave region owing to surface depth occlusion. As we shall see, these asymmetries are important for the details of the luminance CIs.

The predicted outcome under the punctate lighting hypothesis is complicated further by the OS's practice of presenting aerial imagery with geographic north at the top of the image, consistent with most geographic maps. However, in the United Kingdom (UK), the sun shines predominantly from the south. This produces aerial images that are lit from the “south,” in this case, meaning from the bottom of the page/screen. Expert remote sensing surveyors are thus accustomed to viewing aerial images as if lit from below their line of sight, which conflicts with a well-known bias in the population known as the lighting-from-above prior^1^ (e.g. Adams, et al., 2004; Ramachandran, 1988; Sun & Perona, 1998). As the experts have spent many years working with aerial imagery lit this way, we speculated that their natural lighting biases might have diminished, or even switched direction. We made no predictions about whether either of the two lighting assumptions (punctate or diffuse) would dominate in the experiment, accepting we might see both, but under the punctate lighting prior, we expected luminance peaks in the CI to be offset “north” of the perceived center line of hedges for novices, as per the conventional prior, but “south” of them, or with smaller offsets, for the experts. Similarly, under this hypothesis, we expected bi-lobed luminance CIs for the reasons to do with lighting and shading outlined above. More generally, because novices and experts have potentially different priors, we expected the two groups to have different sensitivities to image orientation (lighting direction in the hedge and ditch images) and to have qualitatively different luminance profiles in their classification images.

### Overview and aims

In the work here, we introduced a novel variant of the CI technique designed to provide simultaneous estimation of luminance and disparity templates. We did this for a feature identification task using aerial images to address the research questions introduced above and summarized below as hypotheses. These concern the expected differences between experts and novices and serve to scaffold our results. However, our observations and conclusions extend beyond these a priori expectations.

H1, Utilization of stereoscopic cues: We expected that experts will be better in sampling relevant information from stereoscopic aerial images. This will be shown by greater amplitudes and greater spatial extents of the disparity CIs for experts compared to novices. Further, experts will show greater sensitivity to the stereoscopic profiles of targets, as revealed by their accuracy in categorical ratings.

H2, Lighting direction bias: Compared to novices, experts will have different or diminished lighting direction biases, and, by this token, novices will show a greater tendency to lighting from above in their CIs compared to experts. Further, regardless of their CI structures, experts and novices will show different sensitivities to lighting direction, with novices having a greater tendency to respond according to an assumption of lighting from above.

## Methods

### Visual stimuli and the image generation pipeline

High-resolution aerial-view landscape photographs covering land areas of approximately 2.5 km × 1.5 km were sourced from the OS. Stereogram pairs were created using two images that covered overlapping landscape areas, spaced apart along the aircraft's flight path. Six landscape features were isolated: three landscape features were hedges, found in Cambridgeshire, UK, and three features were ditches, found in Somerset, UK. Features were selected based on the following criteria: (1) the levels of shadow/sunlight were moderate; (2) the features were horizontally aligned within 15° of the aircraft's flight path to facilitate horizontal binocular disparity; (3) the features were of a similar vertical extent and spread across the width of the image segment selected; (4) the features were straight; and (5) the features had usable stereoscopic information (shallow ditches were excluded) as judged by two of the authors.

**Figure 1.**
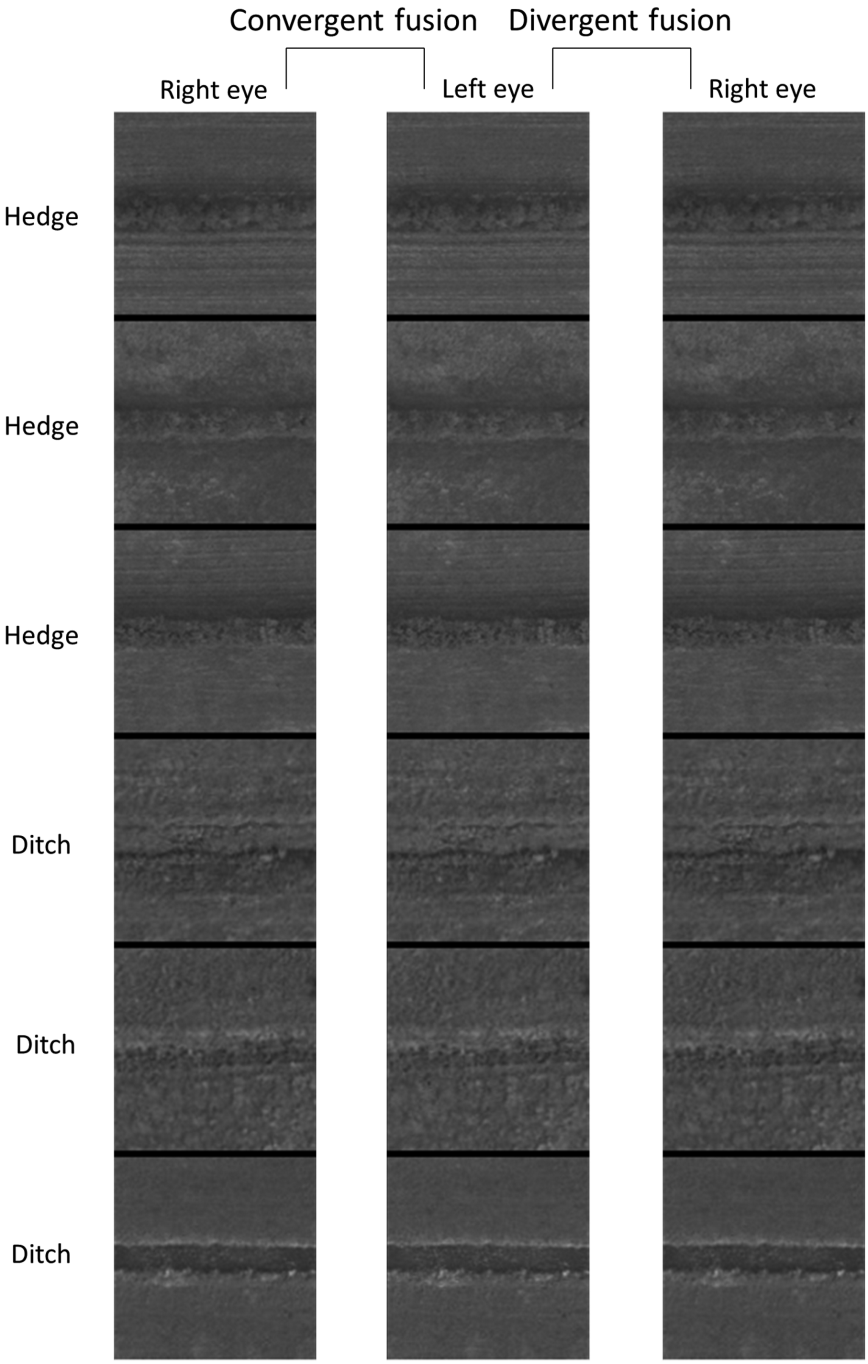
The landscape features used in our stimuli (see Figure 3) after the minor rotation needed to achieve a visually horizontal feature. The top and bottom three pairs are hedges and ditches, respectively. The images are stereogram pairs arranged for free-fusion. These images are shown with geographic north at the top of each image as per OS practice (see text for details). In these images, our terms “north” and “south” refer to the upper and lower halves of the images (and their parts), respectively, and also, to a first approximation, the compass. Notice subtle lighting cues owing to sunlight originating as if from the “south” in these images. © *Crown copyright and database rights 2024 OS*, used with permission.

The six image pairs were processed with MATLAB (MathWorks, Inc.) and Python to create landscape stimuli. Each image was: (1) rotated to horizontal alignment using bicubic interpolation (mean rotation = 7.35° and range = 0–14.5°); (2) resized so that features had the same vertical extent using bicubic interpolation (mean scale factor = 0.85 and range = 0.52–1.2); (3) linearized to undo a compressive nonlinearity applied in the OS image pipeline; (4) converted to grayscale using Equation 1:

(1) $$\begin{array}{c} L=0.2125R+0.7152G+0.0722B,\; \end{array}$$

where *L* = luminance, *R* = red color channel, *G* = green color channel, *B* = blue color channel; (5) cropped to 128 × 128 pixels; and (6) standardized to have the same mean luminance and average root-mean-square contrast as the 12-image set. The images were processed and stored at 16-bit greyscale resolution throughout to prevent losses. These transformations were designed to produce horizontally oriented target features of similar sizes while removing color and luminance variations in the original photographs that may have varied due to the feature types being photographed at different locations, time of year, and time of day. Figure 1 shows the final images used in the study.

#### Introducing the dual-noise test-image for CIs

In a novel step, we imposed both luminance and disparity noise onto our test images allowing the simultaneous estimation of luminance and disparity CIs. A unique white noise texture (range ±1.0, 128 × 128 pixels) with randomly varying, non-zero mean was generated on each trial. This texture was low pass filtered with a first order Butterworth filter with a cutoff frequency of nine cycles per image. The noise texture was then added to the two landscape images in each stereoscopic pair to create noise + feature images (noise and image contrasts were normalized to 35% and 65% of their original contrasts, respectively).

**Figure 2.**
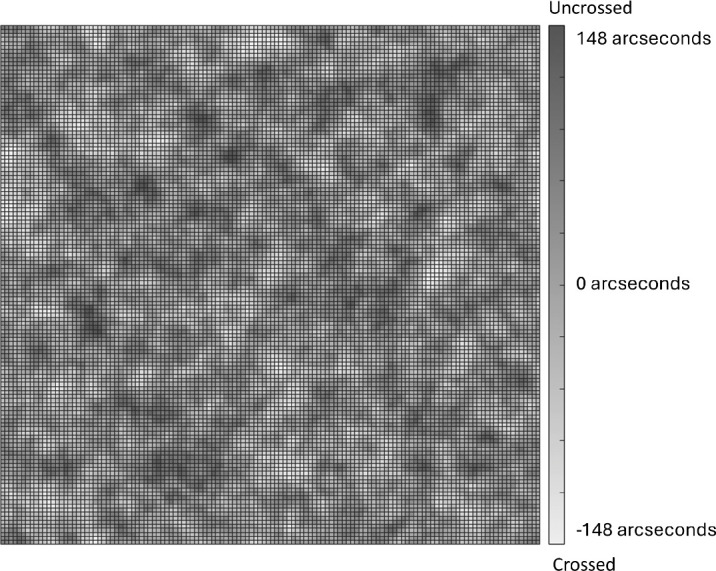
Example Z-coordinate texture used to map random disparities.

Another low pass filtered noise texture (with properties as above) was generated on each trial to create a random disparity map describing disparity offsets (see Figure 2 for an example image). Pixels in the two noise + feature images (one for each eye) were displaced horizontally by an amount determined by the random disparity map, thus adding disparity noise. Each image in the stereo pair bore half the required shift so that the images for the two eyes were transformed equally but in opposite directions. When presented in the stereoscope (described below) this produced a range of 0–296 arcseconds of random disparity (quantized to 8 levels) in the stimulus image pair and required sub-pixel shifts in the position of each pixel in the noise + feature images.

**Figure 3.**
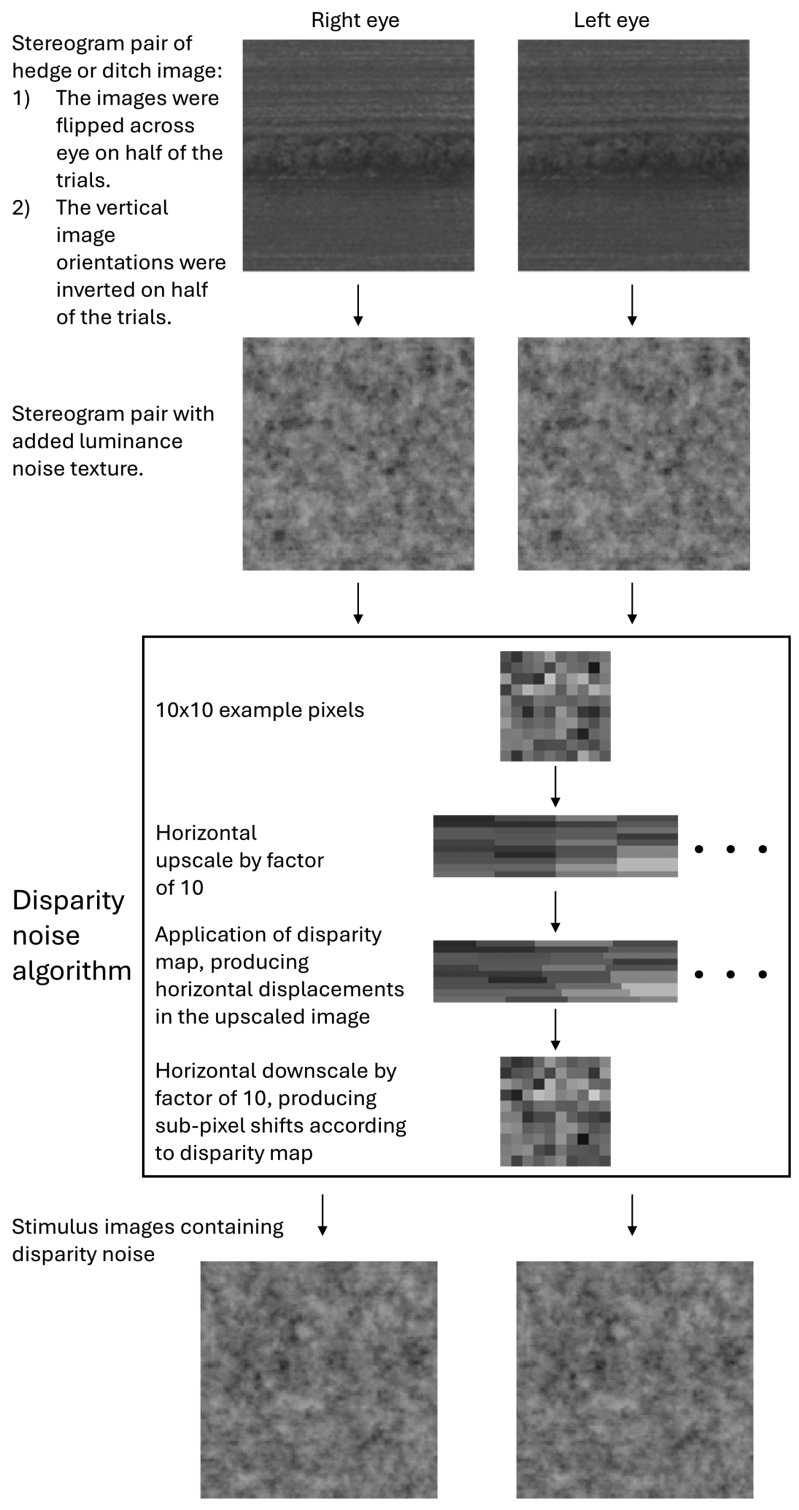
Dual-noise procedure for making stimulus images on each trial using the hedge and ditch images in Figure 1. Paired images are arranged for crossed fusion and with the low signal-to-noise ratios used in the experiment. This means that the cross-fusing reader is unlikely to witness much meaningful signal. See text for further details. Top image pair, © *Crown copyright and database rights 2024 OS*, used with permission.

Figure 3 illustrates the procedure for adding noise to the stimulus images, including the process for producing sub-pixel shifts, which was similar to the one used by Georgeson, Yates, and Schofield (2009). Each noise + feature image was first upsampled in the horizontal direction by a factor of 10 to produce a 128 × 1280-element image. The upsampled luminance elements were then displaced based on values taken from the equivalent location in the disparity map (see Figure 2). The amount of displacement applied varied horizontally across the image meaning that two or more luminance elements in the original image could be displaced to the same location in the transformed image. To address this problem, the competing luminance element that was subject to the least crossed disparity (i.e., the one that would appear furthest from the observer) was discarded and only the element subject to the most crossed disparity (i.e., closest to the observer) was retained. The disparity shifts could also result in gaps where no luminance element was assigned to a location in the transformed image. These gaps were filled with random luminance values sampled from a white noise texture. The image array was then downsampled in the horizontal direction by averaging, thereby recreating the original image resolution. Where determined by the disparity map, this procedure resulted in sub-pixel disparity shifts by virtue of subtle variations of luminance between the two eyes such that the “center of mass” of the grey values comprising features in the stereo pair was subtly shifted in each eye. Note that the random/noisy disparity shifts were applied in addition to the existing disparities between features in the original landscape images. Thus, the original disparities were retained but were heavily distorted by the disparity map analogous to the distortion of the original luminance features by the luminance noise.

Finally, the inverse gamma functions of the monitors were applied to the stereo image pairs to ensure that luminance was linear for our displays. The bottom part of Figure 3 shows an example stimulus pair. Stimulus images were intentionally masked heavily with both luminance and disparity noise because the CI technique benefits from the strong influence of noise on behavioral responses.

#### Disparity and lighting direction

**Figure 4.**
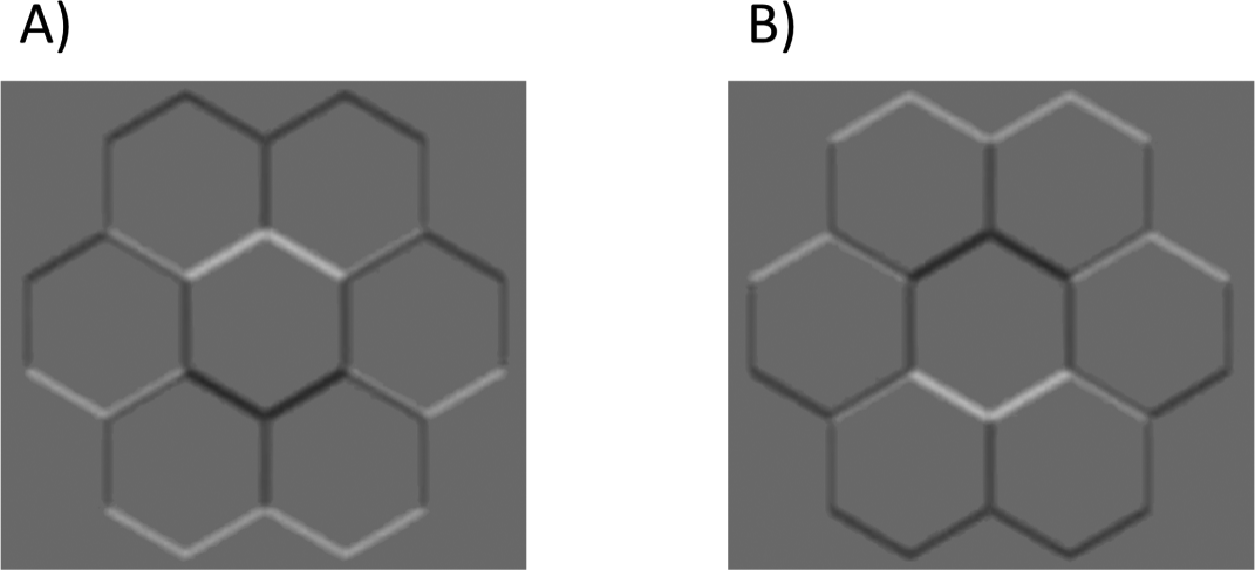
Hexagonal lattices where the light and dark parts of the image provide cues to interpreting 3D relief. (**A**) Is the same as (**B**) but rotated by 180°. These images are included to demonstrate our point about lighting direction and the perception of 3D shape and were not used in the experiment.

Before applying the luminance and disparity noise described above the stimuli were treated in each of two ways. In one manipulation, the landscape images were swapped between the two eyes, so that the disparity of the hedge or ditch was inverted. A hedge image thus changed from having substantially crossed disparity to substantially uncrossed disparity and appeared ditch-like, and vice versa for the ditch images. In a second manipulation, the landscape images were inverted about their horizontal axis, maintaining horizontal disparities but inverting the spatial relations of light and dark image features. In principle, these features can provide cues to 3D relief from highlights and shading. For example, most people report that the middle hexagon in Figure 4a looks like a bump while the same image region in Figure 4b, rotated by 180°, looks like a dimple (Ramachandran, 1988). Underpinning these perceptions is an assumption that lighting comes from above. Therefore, we wondered whether image orientation would also influence the perception of hedges verses ditches.

Each of our two image treatments was performed on a trial-by-trial basis with an independent probability of 50%. This created a stimulus set with an overall 2 × 2 × 2 design (hedge/ditch; correct/inverted disparity; original/inverted orientation).

### Equipment

Participants were seated in a dimly lit and secluded room with their chins on a chinrest in front of a mirror stereoscope. The monitors provided the primary light source in the room, apart from in the testing room in Southampton (see below), where window blinds allowed low levels of diffuse daylight to enter the room. This light applied equally to both viewpoints in the stereoscope. Two front-surface mirrors angled at 45° were mounted 6 cm in front of the participant. These directed images to the observer from two ASUS ProArt PA329C monitors (3840 × 2160 pixels, 710 × 405 mm active screen region) placed on either side of the mirror mount with a total viewing distance of 990 mm. Each monitor pixel subtended 37 arcseconds. Images were scaled in PsychoPy (version 2020.2.10; Peirce et al., 2019) so that a single element from a stimulus image occupied 5 × 5 pixels on the monitors. Thus, images subtended 6.58 degrees of visual angle and the average disparity of the tops of the hedges in pre-noise stimuli was estimated at about 308 arcseconds. Apart from the pre-processing noted above, stimuli were generated and presented using PsychoPy with a modified version of the noise component.

### Participants and ethical considerations

Twelve participants (mean age = 38.7 years and range = 23-62 years) were recruited by targeted email advertisement or direct communication. Participants were categorized as experts or novices depending on their level of experience with remote sensing surveying. An expert was defined as someone with two or more years of experience with remote sensing photogrammetric tasks. A novice was someone with no experience with remote sensing photogrammetry. Six participants were experts (mean age = 43.8 years and range = 23-62 years) and employees at the OS with an average of 8 years of experience (range = 2-20 years). The six novices (mean age = 33.5 years and range = 25-45 years) comprised two non-surveying staff at the OS, one staff member at Aston University, and three PhD students. The eight OS employees were tested at their offices in Southampton, UK, and the other four participants were tested on the Aston University campus, Birmingham, UK. Both groups had an average of 4 years of completed university-level education. No participant was experienced in creating or participating in psychology or psychophysics studies. Participants gave informed consent and were compensated with payment at a rate of £10 an hour. All participants were assured that their data, including screening data, would be confidential and anonymized before discussion between the authors. The project was reviewed by Aston University's College of Health and Life Sciences Ethical Review committee (approval number 1843).

### Screening and exclusion procedure

A screening procedure assessed the eyesight and binocular stereopsis of each potential participant for the experiment. Participants wore their normal optical correction where appropriate. They were tested for standard visual acuity using a Snellen test and undertook the “gold standard” (Garnham & Sloper, 2006) TNO test for stereoscopic vision; based on random-dot-stereograms that provide no monocular cues to the target.

The results of the TNO test are shown in Table 1 and are within normative bounds of a sample of 1058 participants who had a median TNO stereoacuity of 60 arcseconds (Bosten, et al., 2015). No exclusion criterion was set for this test. We will return to Expert 5's relatively high TNO threshold later in the paper but note that Bosten et al. (2015) reported that 8.9% of their sample had a TNO stereoacuity measure of ≥480 arcseconds.

**Table 1.** TNO thresholds for all 12 participants who took part in the experiment.

	TNO threshold, arcseconds	
Participant	Experts	Novices
1	15’’	120’’
2	30’’	60’’
3	30’’	15’’
4	30’’	30’’
5	450’’	15’’
6	30’’	60’’

TNO threshold describes stereoacuity threshold in arcseconds from the TNO test. Participant/observer numbers are nominal and correspond to the participant numbers in the figures below.

Participants were familiarized with the stereoscope by observing 10 images that contained either a “flat” texture or a stereoscopic texture with a square target defined by crossed disparity. Participants then carried out a discrimination task (40 trials) where a central disparity-defined square (740 arcseconds of disparity and side length 1.44 degrees of visual angle) had either crossed or uncrossed disparities. The task was to report whether the square was in a “near” or “far” depth plane compared to the surround. Responses were made by pressing a button on a keyboard. Participants had to score above 90% correct to pass this test. Those who failed were thanked for their time and given £5. The 12 participants described above passed this screening test, but this test led to the exclusion of two other novice participants and no experts.

### Experimental procedure

#### Preliminary procedure: General familiarity

To familiarize all participants with the concept of aerial stereoscopic imagery, they were shown the same ground view photograph of two houses followed by an OS aerial stereogram pair of the same houses viewed through the stereoscope. They were told that these were different views of the same scene, the second one from above, and that they would be viewing aerial images containing stereoscopic depth like the houses but showing hedges and ditches. Participants were then shown ground-view images of a hedge and a ditch and told they would be looking for these features but from an aerial perspective. They were not shown any aerial-perspective images of hedges and ditches as part of the familiarization procedure, and they were not told how many different images they would be shown.

#### Preliminary procedure: Familiarity and instructions

At the start of their first experimental session, participants practiced for approximately 20 trials under the supervision of author E.S. following the main procedure below. They were instructed to press the left and right arrow keys on a keyboard for “ditch” and “hedge” responses, respectively. They were told that the task would often feel very difficult, but they should make their best estimates to find hedges and ditches. They were also told that various hedge and ditch stimuli would be presented, and that these were always in the same location and had the same size. The author gestured with his hand to highlight the shape outline and the size of the hedges and ditches over the monitor.

To ensure appropriate vergence control, between each trial a black fixation cross was presented in the center of the screen. The vertical bar of the cross was split across the two eyes. To achieve good convergence, participants were instructed to fuse the cross to make it appear “complete,” like a “+.” If the cross appeared to drift apart, participants were instructed to close their eyes or look away for a moment to “reset” their convergence and on returning attention to the display, to wait until the cross appeared fused before making their response which would also start the next trial. To further aid fusion, a high contrast border featuring white rectangles on a black background surrounded the image presented to each eye.

#### Main procedure

Stimuli were presented for 750 ms and participants were allowed unlimited response time. No feedback was provided. A response triggered a new trial after a 630 ms delay. The high contrast border surround and the fixation cross were always present, except the fixation cross was removed when the stimuli were displayed.

The stimuli were presented with congruent or incongruent disparities and original or inverted orientation (see above) with equal probability on each trial. The third stimulus factor (original target feature = hedge or ditch) was blocked (i.e., a block of trials contained either only original hedges or only original ditches) but participants were not informed of this. This was done because there were differences between the two sets of images (e.g., time of day/year, geographic location, and isolated grassland features) all unconnected but correlated with the intended feature of interest. By blocking stimuli in this way, we ensured that such extraneous features could not influence decisions within each block. (We discuss the implications of this in a later subsection.) The disparity reversals applied within each block ensured that the stimuli were presented as hedge-like and ditch-like with equal probability on each trial regardless of the block type. Sessions alternated between block type with the starting order counterbalanced across participants. Each session contained a single block of 500 trials and there were 20 sessions lasting about 15 minutes each giving a total of 10,000 trails per participant. Breaks were permitted between sessions and sometimes this was overnight. Eleven participants completed the experiment over 3 days, for one participant, it took 4 days. The total experimental time for each participant was about 6 hours.

Following their final session, participants were asked to describe what they had been looking for when deciding whether stimuli were hedges or ditches. Participants were also asked whether they were aware of sunlight and/or shading influencing their decisions.

## Results and discussion

### Debriefing

Expert 3 and Novices 2 and 4 reported using a specific luminance strategy where hedges and ditches were assumed to be light and dark, respectively (i.e., a “dark is deep” strategy). The other five experts and four novices reported using stereoscopic depth as a primary strategy. These experts also stated that when luminance cues were used (consistent with “dark is deep”) this was usually as a secondary strategy. No participant mentioned that sunlight direction or shadow location influenced their decisions when asked about the direction of the light source. This suggests that participants were unaware of using a punctate lighting assumption. No participant mentioned the two block types.

### Organization of main results

In the main, we deal with issues around disparity first followed by those around lighting and luminance, starting with CIs, progressing to categorical details, then returning to CIs. However, we begin with some overall observations of the CIs then describe how we quantified them before turning to interpretation and our hypotheses.

### Classification images and informal observations

**Figure 5.**
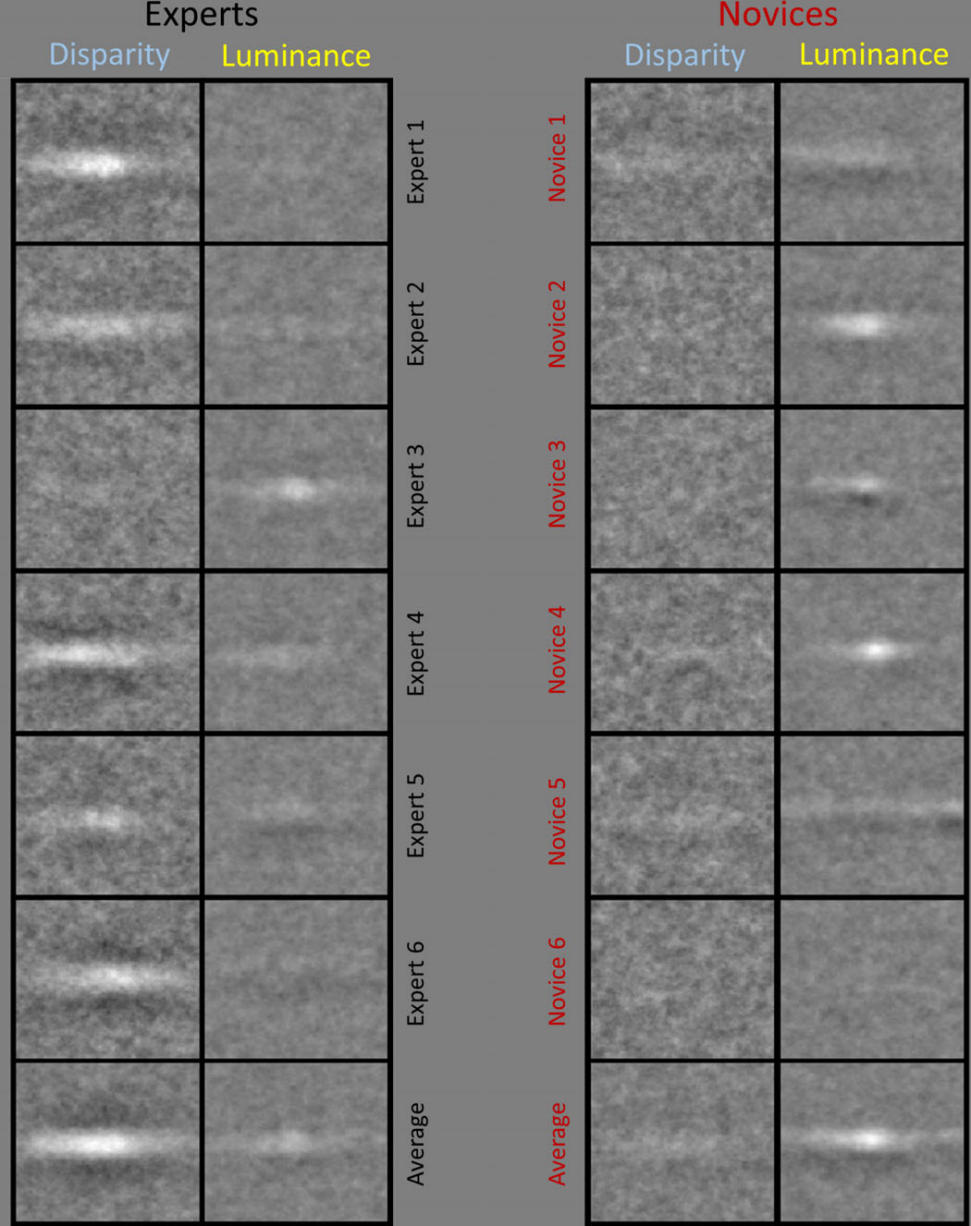
Classification images (CIs) for disparity and luminance where “ditch” response textures were subtracted from “hedge” response textures. For both types of classification image, lighter and darker pixels represent mounds and troughs, respectively. Thus, for the disparity CIs, the light regions derive from observers responding “hedge” and “ditch” when there were crossed and uncrossed disparities in those image regions, respectively. For the dark regions, the disparities were uncrossed and crossed, respectively. Similarly, for the luminance CIs, the light regions derive from observers responding “hedge” and “ditch” when there were light and dark pixels in those image regions, respectively. For the dark regions, the pixels were dark and light, respectively. Observer/participant numbers are nominal. The bottom row of the figure presents group-average CIs.

Luminance CIs were generated from the original noise textures before the addition of the signal images and the application of disparity noise algorithm. Disparity CIs were generated from the Z-coordinate maps for the disparity texture noise textures, without signal (see Figure 2), where light pixels represent crossed (“near”) disparity, and dark pixels represent uncrossed (“far”) disparity. (Our convention here means that the CI grey levels relate to implied 3D relief in the same way for both types of CI.) For each participant, noise textures for luminance and disparity were tagged according to the “hedge” or “ditch” response on each trial and compound images were generated by summing the images for each tag. To generate a CI from all 10,000 trials, “ditch” response compounds were subtracted from “hedge” response compounds (Ahumada 1996; Murray, 2011). Figure 5 shows CIs for each of the 12 participants revealing individual decision templates for luminance and disparity. Some implications of this analysis are considered in the Supplementary file.

Experts produced stronger CIs for disparity than luminance, whereas this was the other way around for novices. This suggests that the two groups used different classification strategies. The center regions of the disparity CIs in Figure 5 are white, indicating that crossed disparity appeared hedge-like and uncrossed disparity ditch-like, as to be expected. Individual differences in lighting assumptions are observed in the luminance CIs consistent with the lighting bias hypothesis (H2), as follows. For some participants, the template centers are white, indicating that lighter patterns appeared hedge-like and darker patterns ditch-like, consistent with a diffuse lighting assumption in shape from shading (e.g., Expert 3 and Novices 2 and 4). For other participants, the luminance CIs show centrally offset positive and negative peaks, consistent with the influence of a punctate lighting assumption on the identification of hedge-like and ditch-like stimuli (e.g., Novices 1, 3, and 5). We provide further discussion of individual differences below.

From casual inspection of the partial CIs from each of the six original hedge and ditch images (see Figure 1), no systematic differences were observed across the different images (results not shown), confirming that participants were consistent in their use of visual strategies across the six images.

### Quantifying the CIs

**Figure 6.**
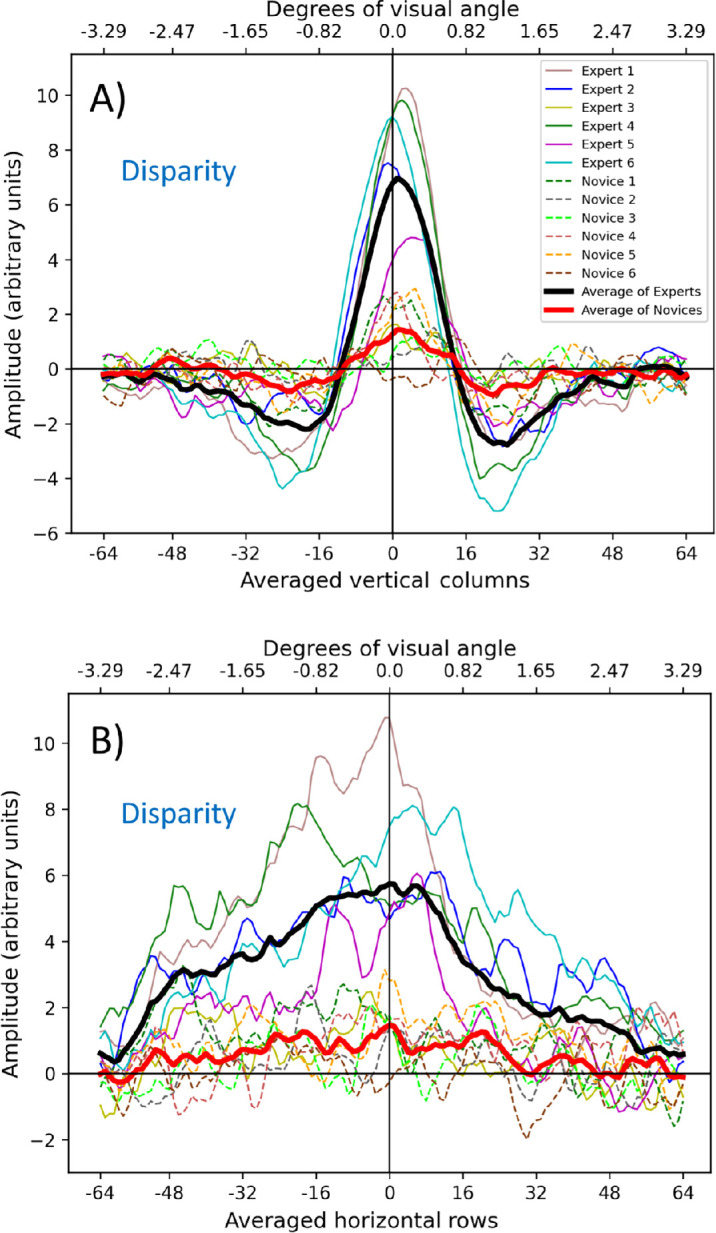
Cross-sections of disparity classification images. (**A**) Vertical cross-sections. Left of center corresponds to “north” in the hedge and ditch images. (**B**) Horizontal cross-sections of the central 20 rows. Experts and novices are shown by solid and dashed curves, respectively. Different colors are for individual observers; thick curves show group averages.

**Figure 7.**
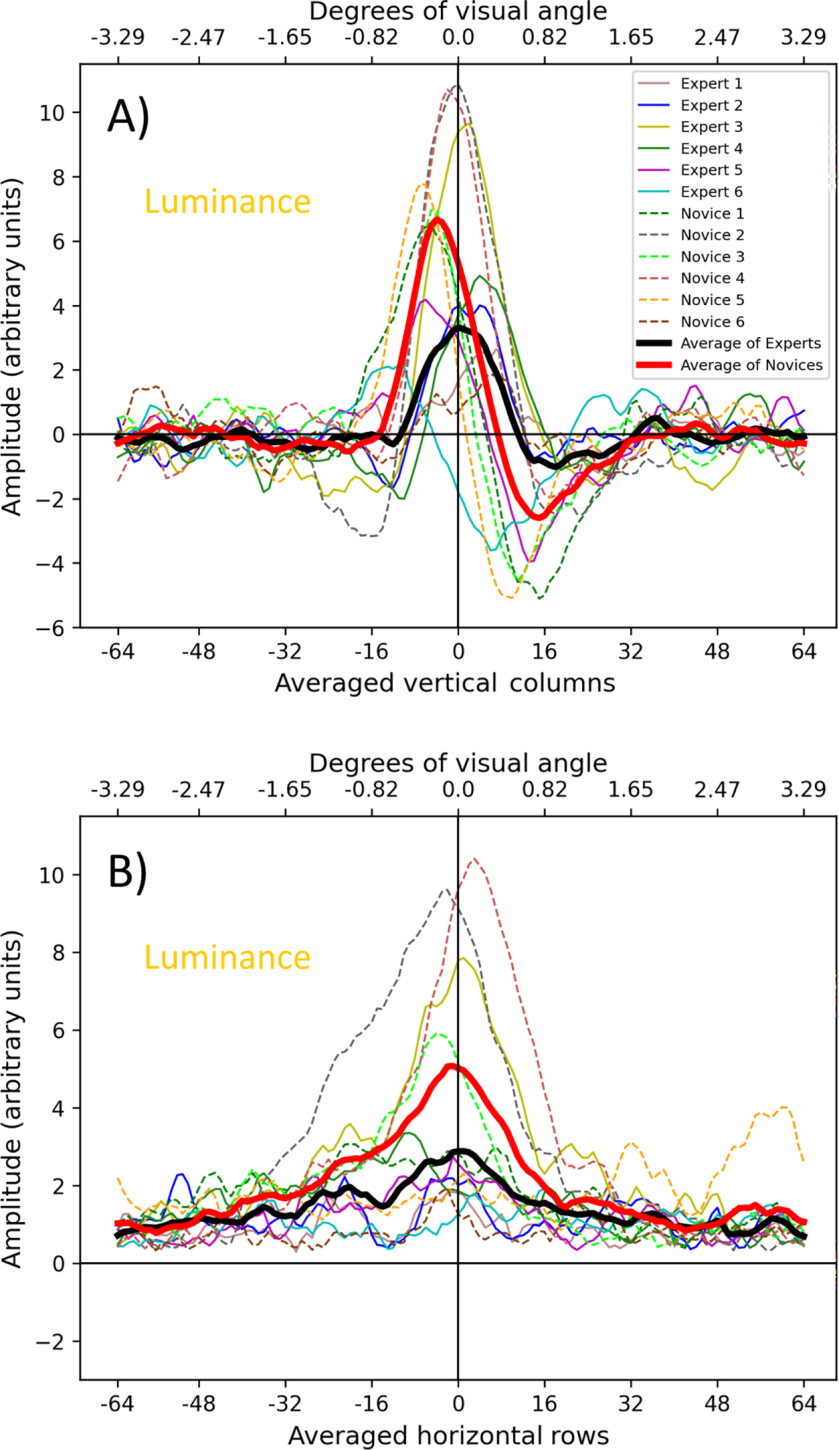
Cross-sections of luminance classification images. The details are as for Figure 6. (**A**) Vertical cross-sections. (**B**) Horizontal cross-sections of the central 20 rows after full-wave rectification (see text for further details).

To facilitate quantitative visualization of the differences between the experts and novices, we plotted cross-sections of the CIs for disparity (Figure 6) and luminance (Figure 7). Figure 6a shows the vertical cross-sections produced by averaging all the pixel columns in the disparity CIs (recall that the target features were horizontal) and has a profile like a Difference of Gaussians with a central positive lobe and two flanking negative lobes (sometimes called a “Mexican hat”). Figure 6b shows the horizontal cross-sections produced by averaging the 20 central rows of pixels (±10 from the center) corresponding with the target location. Only this central region was used for the horizontal cross-sections to avoid cancellations from the outer regions (see Figure 6a) with opposite sign. Figure 6b reveals a curved profile with a peak in the center of the CI. The dashed and solid curves in Figure 6 are for the novices and experts, respectively, and illustrate large group differences for disparity.

The treatment of the luminance CIs in Figure 7 is similar to that for the disparity CIs in Figure 6, but the outcome is different. Figure 7a shows that all observers have distinct positive lobes and several also have negative lobes but often weighted more heavily on one side than the other. Furthermore, while several observers have central peaks, others have peaks offset from the center, the most prominent of which are to the left. This corresponds with “north” (up) in the stimuli, although, in some cases, the offset is in the other direction. These differences necessitated special treatment for the averaging in Figure 7b. As the negative lobes in Figure 7a were offset for some observers, they were prone to cancel the positive lobes when averaged across the central 20 rows. Therefore, to preserve amplitude, these rows were full-wave rectified before averaging.

With the cross-sections defined as above, we characterized them by fitting Gaussian (Equation 2) and Gabor (Equation 3) functions to the horizontal and vertical cross-sections, respectively:

(2) $$\begin{array}{c} f\left(x\right)=A\exp\left(-\frac{{\left(x-\mu \right)}^{2}}{2\sigma^{2}}\right),\; \end{array}$$

(3) $$\begin{array}{c} f\left(y\right)=A\exp\left(-\frac{{\left(y-\mu \right)}^{2}}{2\sigma^{2}}\right)\cos\left(2\pi \frac{y}{\lambda }-\psi \right),\; \end{array}$$

where *x* and *y* are column and row numbers (in pixels units, with 0 in the center), *A* is amplitude, *µ* is spatial offset (in pixels), *σ* is spread (standard deviation in pixels), λ is wavelength (in pixels), and *ψ* is the absolute phase offset (in radians) of the co-sinusoidal component of the Gabor function. Absolute phase offset (*ψ*) was subtracted rather than added (Equation 3) to harmonize the signs between spatial and absolute phase offsets. For convenience, absolute phase offsets were converted to pixel units, *ψ_pix_* (Equation 4):

(4) $$\begin{array}{c} \psi_{pix}=\lambda \psi /2\pi ,\; \end{array}$$

This makes the absolute phase offset parameter, *ψ_pix_*, directly comparable to the spatial offset of the Gaussian, *µ*. Absolute phase (*ψ*) changes the peak position of the cosine component, and spatial offset (*µ*) changes the peak of the Gaussian envelope. The asymmetry of the Gabor function depends on the relative values of these two offsets. This was captured by a relative phase parameter (*φ _pix_*), derived by subtracting the spatial offset (*µ*) from absolute phase (*ψ_pix_*) and converted back to radians to give, *φ*. Thus, *µ*, expresses the lateral shift of the entire Gabor function and *φ* (and *φ _pix_*) expresses the phase shift relative to this. Curve fits were estimated using MATLAB's Curve Fitting ToolBox.

Equation 2 (the Gaussian) has three free parameters (*A*, *µ*, and *σ*) and Equation 3 (the Gabor) has five—the same as the Gaussian plus *λ* and *ψ* (or *ψ_pix_*), or alternatively, *λ* and *φ* (or *φ _pix_*). In addition to these parameters, the location of the Gabor peak (which depends on *µ*, *σ*, and *φ _pix_*) was determined using a MATLAB implementation of the Nelder-Mead simplex algorithm to find the lateral position, *P*, of the function maximum.

**Figure 8.**
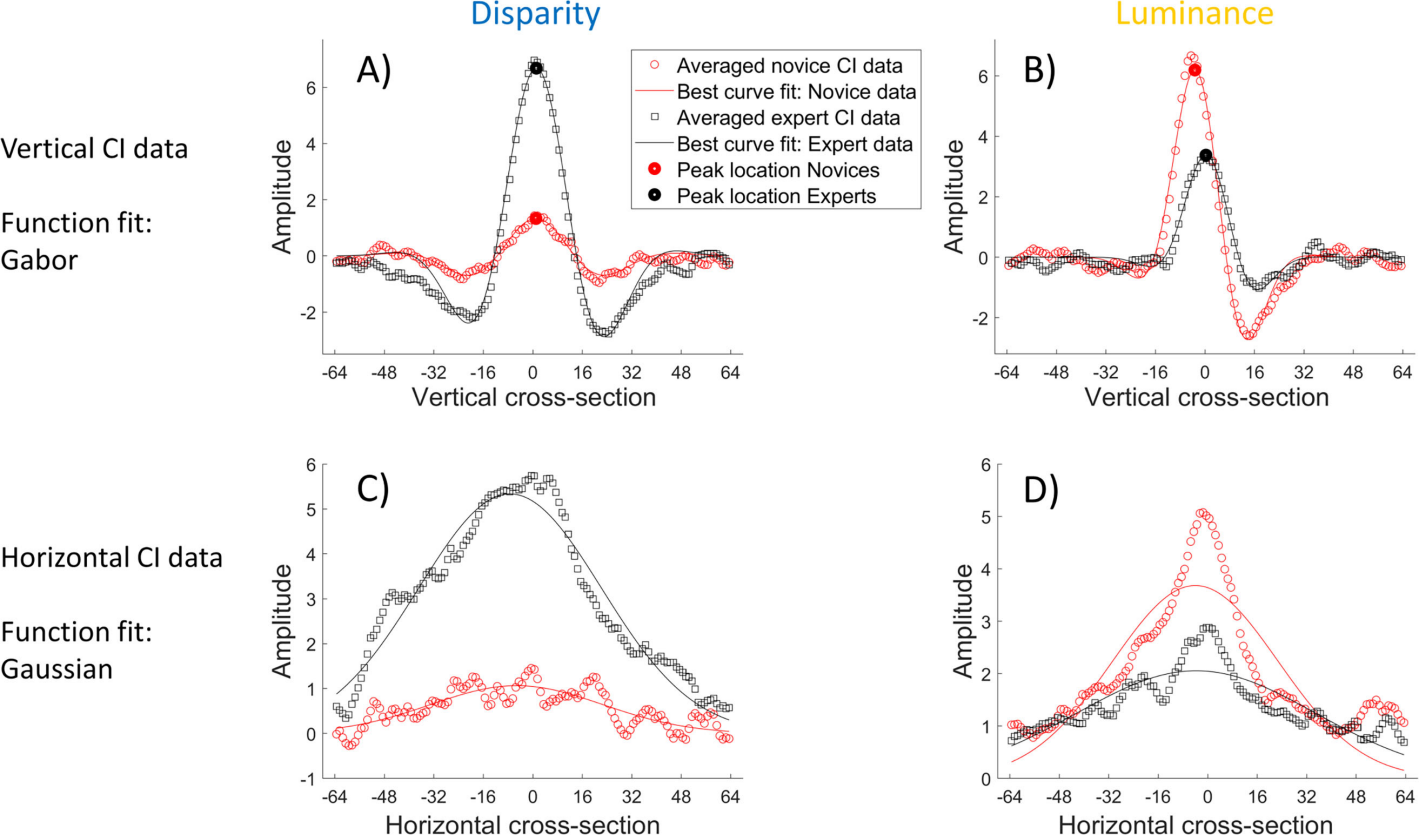
Fits of descriptive curves to the group averaged cross-sections of the CIs (see Figures 6 and 7). (**A**) Vertical disparity results fitted by Gabor functions. (**B**) Vertical luminance results fitted by Gabor functions. (**C**) Horizontal disparity results fitted by Gaussian functions. (**D**) Full-wave rectified (see text for details) horizontal luminance results fitted by Gaussian functions. The origin of the x-axis is the stimulus center. In the top row, left-to-right along the x-axis corresponds with top-to-bottom in the vertical CIs.

The fits of Equations 2 and 3 to the group averages from Figures 6 and 7 are shown in Figure 8, and their parameter values are reported in Tables 2 and 3, respectively. The Gabor fits to individual observers are shown in the Appendix for the disparity and luminance CIs, respectively. Our aim was to use whichever of the parameters above served us best in evaluating our hypotheses. As we shall go on to illustrate, these proved to be: *A*, *φ, P*, and *σ*. By comparison, * λ* and * ψ*, did less to distinguish between the factors of interest. They are included in Tables 2 and 3 for completeness but we do not consider them further. The spatial offset of the Gaussian, *µ*, was arguably more valuable, but for the vertical cross-sections it was subsumed by *P*. For the horizontal cross-sections, *µ* was always significantly negative meaning the fitted Gaussians were shifted a little to the left. We have no explanation for this but given the variability of the data around the fitted profile (see Figures 8c, 8d) we think the observation conveys little or nothing of value and do not consider *µ* any further.

**Table 2.** Gaussian parameters (Equation 2) for fits to the group average horizontal CIs (see Figures 8c, 8d).

	Fitted Gaussian parameters to horizontal disparity CIs (with 95% confidence bounds)		Fitted Gaussian parameters to horizontal luminance CIs (with 95% confidence bounds)	
Group	Experts	Novices	Experts	Novices
Amplitude *A*	**5.33 (5.19 to 5.48)**	**1.07 (0.98 to 1.15)**	**2.06 (1.96 to 2.15)**	**3.68 (3.46 to 3.90)**
Spread σ	29.58 (28.61 to 30.55)	27.78 (25.1 to 30.47)	**38.63 (35.98 to 41.27)**	**26.82 (24.9 to 28.75)**
Spatial offset *µ*	−7.35 (−8.26 to −6.43)	−5.21 (−7.79 to −2.62)	−3.74 (−5.92 to −1.56)	−4.02 (−5.89 to −2.14)
Adjusted *R^2^*	0.937	0.663	0.682	0.715

Non-overlapping confidence intervals between groups are shown in bold. (Confidence intervals were estimated directly from the data with MATLAB's “confint” function). Goodness of fit is shown by adjusted *R^2^*. Parameter values that belong to the x-axis in the figures (σ,  *µ*) are in pixel units. Negative spatial offsets indicate leftward lateral shifts of the peaks in Figures 8c and 8d.

**Table 3.** Gabor parameters (Equation 3) for fits to the average vertical CIs (see Figures 8a, 8b).

	Fitted Gabor parameters to vertical disparity CIs (with 95% confidence bounds)		Fitted Gabor parameters to vertical luminance CIs (with 95% confidence bounds)	
Group	Experts	Novices	Experts	Novices
Amplitude, *A*	**6.69 (6.44 to 6.95)**	**1.33 (1.24 to 1.42)**	**3.55 (3.37 to 3.73)**	**6.65 (6.41 to 6.9)**
Spread, σ	**17.59 (16.84 to 18.33)**	**20.49 (18.85 to 22.13)**	10.64 (9.95 to 11.34)	11.56 (11.05 to 12.07)
Spatial offset, *µ*	2.19 (1.32 to 3.06)	0.92 (−0.8 to 2.64)	**3.02 (2.17 to 3.88)**	**0.44 (**−**0.12 to 0.1)**
Wavelength, *λ*	53.1 (51.94 to 54.26)	50.32 (48.7 to 51.95)	48.08 (46.08 to 50.09)	46.76 (45.52 to 48)
Absolute phase, *ψ_pix_*	0.89 (0.60 to 1.18)	1.03 (0.56 to 1.51)	−**1.07 (**−**1.51 to** −**0.63)**	−**4.78 (**−**5.01 to** −**4.55)**
Absolute phase, *ψ* (in radians)	0.03π (0.02π to 0.4π)	0.04π (0.02π to 0.6π)	−**0.04π (**−**0.06π to** −**0.03π)**	−**0.20π (**−**0.21π to** −**0.19π)**
Relative phase, *φ_pix_*	−**1.3 (**−**1.59 to** −**1.01)**	**0.11 (**−**0.36 to 0.58)**	−**4.09 (**−**4.53 to** −**3.65)**	−**5.22 (**−**5.44 to** −**4.99)**
Relative phase, *φ* (in radians)	−**0.05 π (**−**0.06 π to** −**0.04 π)**	**0.0 π (**−**0.01 π to 0.02 π)**	−**0.17 π (**−**0.19 π to** −**0.15 π)**	−**0.22 π (**−**0.23 π to** −**0.21 π)**
Peak location, *P*	1.13	1.02	0.31	−3.26
Adjusted *R^2^*	0.966	0.898	0.954	0.977

Bold text shows non-overlapping confidence intervals between groups. Goodness of fit is shown by adjusted *R^2^*. Several of the function parameters that relate to the x-axes in the figures (σ,  μ, *λ_pix_*, and *φ_pix_*) are in pixel units, as is the (lateral) peak location (*P*). Negative spatial offsets and phase indicate lateral shifts to the left.

### Interpreting disparity CIs

The amplitude (*A*) of the average disparity CIs (Figure 8) was about five-times greater for the experts than for the novices (left of Tables 2 and 3; red and black curves in Figures 8a, 8c), confirmed by an independent samples *t*-test (Welch's *t*(5.59) = 3.79, *p* = 0.005; one-tailed). However, for the vertical cross-sections (see Table 3), the Gabor spread (*σ*) for the novices was slightly greater than for the experts, although no reliable differences for the Gaussian spreads (*σ*) of the horizontal cross-sections were found (see Table 2). Thus, the disparity CIs suggest that experts were better than novices at using disparity cues for depth (H1), although they did not sample this information over a greater spatial extent than novices.

**Figure 9.**
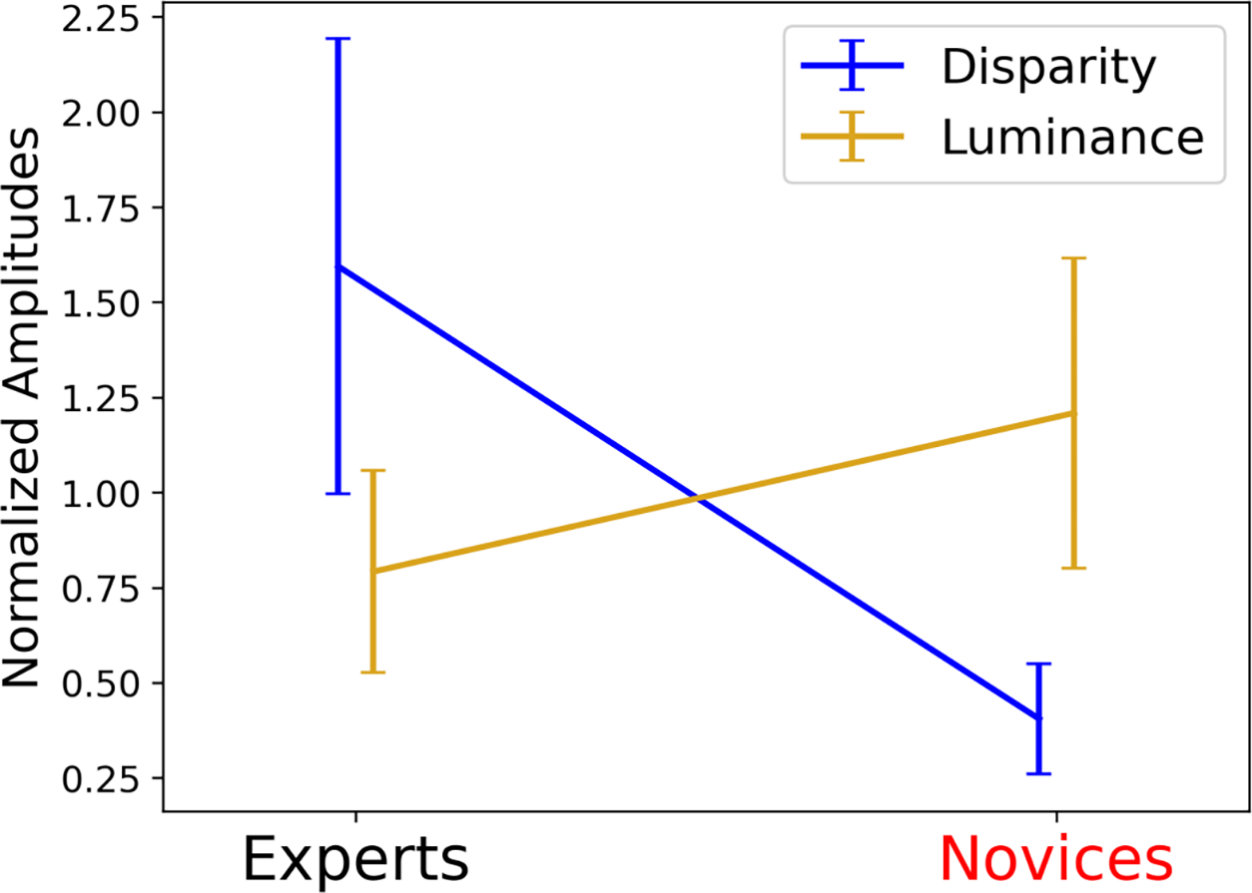
Interaction of CI amplitudes across cue type and groups. The amplitude, *A*, for each of the 12 participants (see Appendix) was normalized for each noise type (disparity and luminance) by dividing by their relevant mean. Error bars are ± 95% confidence intervals.

Both groups reportedly attempted to use disparity cues (see Debriefing, above) but experts prioritized them over luminance cues, particularly by comparison to novices. Seeing this in the group fits (see Table 3) is problematic because we have no measure of noise equivalence across noise type (disparity and luminance) and cannot derive signal amplitude-to-noise ratios to make the relevant comparisons. We addressed this by dividing the amplitudes from the fits to the 12 individual observer results (see Appendix) by their mean for each noise type. A two-way repeated measures ANOVA (Group = expert and novice; Cue type = disparity and luminance) on these normalized results revealed a significant interaction between participant group and cue type (*F*(1, 5) = 15.61, *p* = 0.011)^2^: on average, experts prioritized disparity over luminance but this was the other way around for novices (see Figure 9). Note also that the luminance amplitudes (*A*) for the novices were higher than for the experts (see Tables 2, 3). This means the expert superiority with disparity cannot be attributed simply to greater engagement with the task since under that account, we would not expect novices to outdo experts on luminance cues.

Notably, the CI disparity difference for experts and novices did not derive from stereoacuity because the two groups did not differ in their TNO test scores (see Table 1; one-way ANOVA; Welch's *F*(1, 5.53) = 0.430, *p* = 0.538). When Expert 5 was removed as an outlier (see Table 1), the difference remained nonsignificant (Welch's *F*(1, 5.34) = 1.93, *p* = 0.220). Furthermore, Expert 5 had a TNO threshold (450 arcseconds) 15 times higher than the median and modal TNO threshold in our sample (30 arcseconds) but produced a higher contrast disparity CI than any novice nonetheless (see Figure 6a). A further observation is that the side-lobes in the disparity templates were more apparent for experts than for novices (Figures 6a & 8a). This shows that with experience, decisions about a feature's stereoscopic profile are influenced more by the surrounding context.

### Signal detection analysis (*d′* and bias)

The sensitivity measure, *d′*, is a bias-free estimate of an observer's sensitivity to signal under the tenets of signal detection theory (Green & Swets, 1966; Macmillan & Creelman, 2004). In a traditional yes/no procedure, the signal is the stimulus the observer is trying to detect, and the ground truth is whether the stimulus was presented. In our CI experiment, trials were single intervals and always contained a stimulus feature—either a hedge or a ditch. Nonetheless, for our stimulus-response task, we are free to assign either one of these (e.g., hedges) to the target category, and record hits (hedge-hedge), misses (hedge-ditch), false alarms (ditch-hedge) and correct rejections (ditch-ditch) in the conventional way to derive *d′*^3^. However, our stimuli were counterbalanced in a 2 × 2 × 2 design for image factor, so what defines the signal among the eight different possibilities? To address this, we used *d′* analysis in an inventive way to investigate whether the categorization of our stimuli as hedge-like or ditch-like was influenced by each of our three image factors/manipulations. This required defining the signal in our analyses in each of three ways: (1) luminance image content across the photographs (physical hedge or ditch), (2) binocular disparity (crossed (convex) or uncrossed (concave)), and (3) lighting highlights and shadows (consistent with the hedge or ditch relief associated with lighting from above; see Figure 4). Therefore, in (1) a hit was recorded if the response was “hedge” and the image contained the original visual texture of a hedge. In (2) a hit was recorded if the response was “hedge” and the image contained convex binocular disparities consistent with a hedge. Importantly, in (3) a hit was recorded if the response was “hedge” and the stimulus was oriented such that the lighting produced a highlight above the image centerline and a shadow below the image centerline (because of the southerly lighting direction, this was the case for correctly oriented ditches and spatially inverted hedges). Note that in (2) and (3) the ground truth for hedge (and ditch) no longer relates to whether the image was a photograph of a hedge or a ditch, but to binocular and monocular cues for 3D relief, respectively. In addition, note that for all three analyses, the status of the two factors excluded from the definition of ground truth were irrelevant for determining hits, misses, false alarms, and correct rejections.

We performed our analysis according to each of our three assumptions about ground truth to reveal the ground truths assumed by our observers in their task.

#### Bias

**Figure 10.**
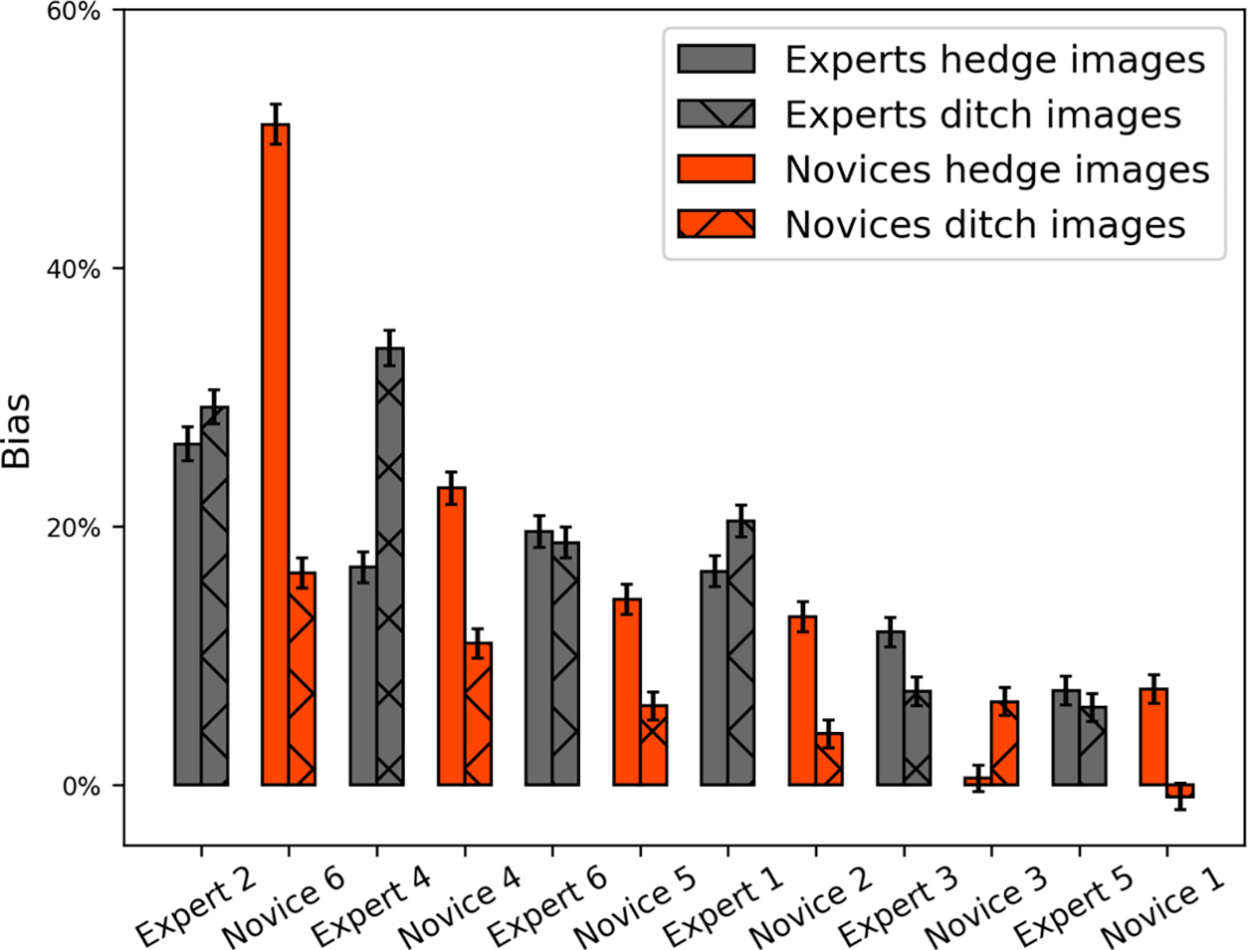
Individual biases (100 × (*n* “hedge” responses - *n* “ditch” responses)/(*n* “hedge” responses + *n* “ditch” responses)). All observers made more “hedge” responses than “ditch” responses. Different shading shows the analysis split by the experimental blocking of trials (into stimuli containing greyscale images of hedges and ditches). Observers are rank ordered by bias (averaged across blocks) within group (left to right). Error bars show 95% confidence intervals (Clopper-Pearson method).

The analysis of bias was common for all three assumptions of ground truth since it deals only with the two response categories (“hedge” and “ditch”). The results are shown in Figure 10 for each observer where the bias measure (expressed as percentage [%]) is given by the number of “ditch” responses subtracted from the number of “hedge” responses and normalized by the total number of stimuli. For both types of experimental block, all observers were biased toward hedges, in some cases, quite strongly (e.g., a bias of 30% indicates a 65:35 split for hedges:ditches), consistent with the well-known convexity bias (Adams & Elder, 2014; Champion & Adams, 2007; Langer & Bülthoff, 2001; Perrett & Harries, 1988)^4^. From our bias measures alone, we cannot determine the relative proportions of response bias and perceptual bias, but a 3D relief matching task (Liu & Todd, 2004) points to a perceptual origin for the convexity bias and the consistency of our results with this suggests that perceptual bias was involved. We consider this no further (bias has no bearing on our hypotheses), but we note that the presence of bias demands a bias-free measure of sensitivity (*d′*), rather than percent-correct.

#### Initial observations of d′ analysis

**Figure 11.**
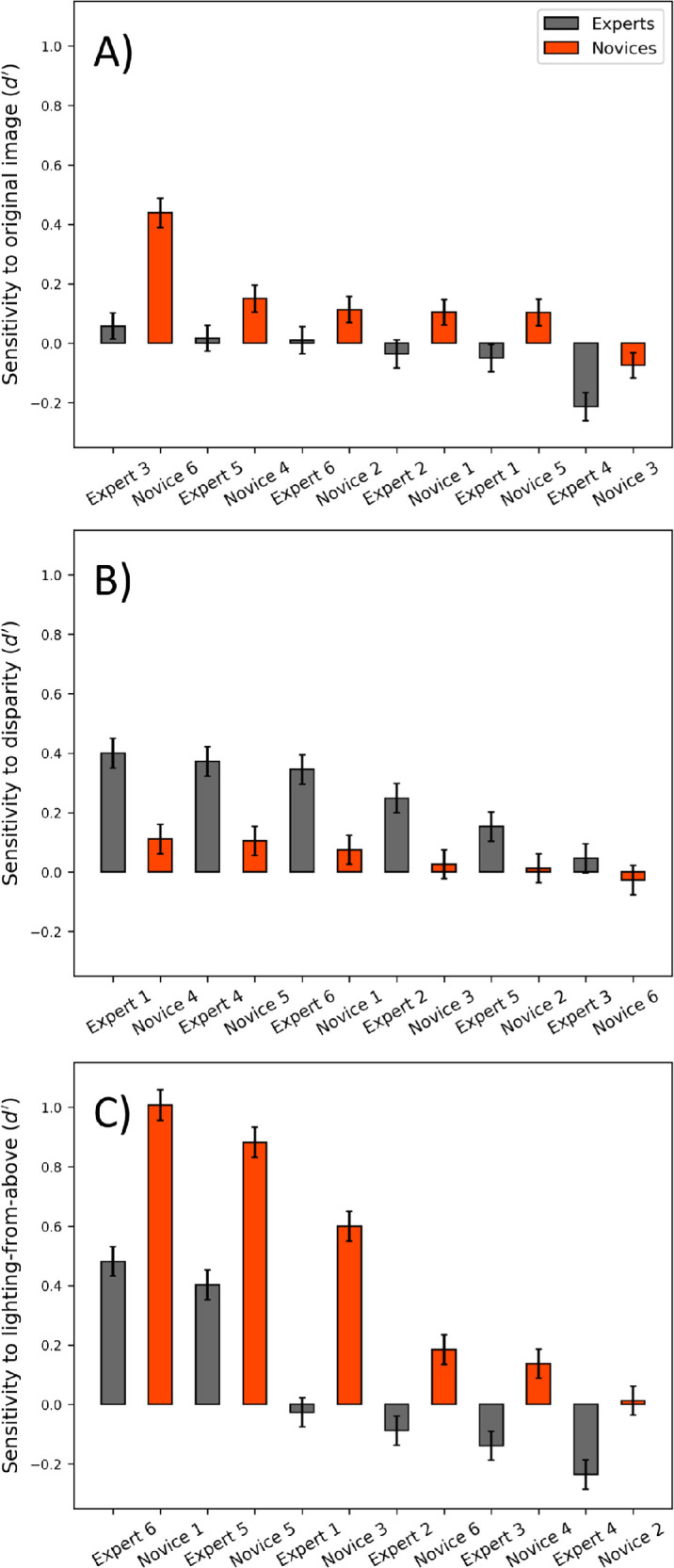
Individual sensitivities (*d′*) to: (**A**) original image, (**B**) disparity profiles, and (**C**) lighting from above. Observers are rank ordered within group (left to right) according to their sensitivity in each plot. Error bars show 95% confidence intervals (Macmillan & Creelman, 2004).

Figure 11 shows sensitivity (*d′*) for each observer where the ground truth was set by (a) the greyscale image content, (b) the sign of binocular disparity, and (c) an assumption of lighting from above (all prior to the addition of noise). In (b) and (c), the consistency of signal sensitivity across image class was typically high (not shown) and the results were collapsed across block type. In (a), the results were necessarily collapsed across block type to determine *d′*; see the next subsection for details. Our first observation is that sensitivity measures varied with the assumption about ground truth, with the grayscale image content (a) being the least valuable to observers in general. Novices (red) systematically outperformed experts (dark grey) in this respect, but with sensitivities so close to zero we are reluctant to consider this further. (See also the next subsection). The higher sensitivity of Novice 6 is notable, although it poses something of a puzzle. This participant is generally an outlier, having the strongest bias overall (see Figure 10)—which might go some way to explaining the absence of structure in their CIs (see Figure 5)—but little or no sensitivity to the other factors for ground truth more commonly used by the other participants (see Figures 11b, 11c). We consider this observer further in the next subsection.

#### Blocking and an assumption about observer stationarity: Not a serious concern

A common assumption in visual psychophysics is that the observer is stationary—that their sensitivity does not vary over the experimental period. In contrast detection, this is a reasonable assumption because empirical estimates show that non-stationarity across typical experimental blocks is similarly low in magnitude to the variability of within-block threshold estimates from undersampling (Wallis, Baker, Meese, & Georgeson, 2013). Our calculations of *d′* involved collapsing data over all blocks of trials. When the ground truth was derived from disparity or lighting direction, trials were interleaved within blocks, and our assumption of stationarity is fairly safe. However, our trials were blocked for greyscale images of hedges and ditches, so might our *d′* analysis (see Figure 11a) for this (ultimately, least interesting) situation be undermined by a switching of strategy (a non-stationarity) between blocks, perhaps prompted by a confounding cue (e.g., the striations in the first and third rows of Figure 1)? We think this is unlikely to be a serious problem for the following reasons.

First, none of our observers expressed knowledge of the blocking across trials.

Second, the combination of (i) our low signal-to-noise ratios and the relatively small size of the target features, compared to their surrounds and (ii) the a priori unlikeliness of this blocking arrangement given that participants were always asked to judge between hedge and ditch in each block, makes this seem an unlikely strategy.

Third, if responses were systematically biased across blocks, this would translate to high magnitudes of *d′* (the sign depending on whether the observer's assumption about block identity was correct or incorrect). But for the stimulus factor in question, these were typically close to zero (see Figure 11a).

Fourth, even if such a strategy played a minor role, perhaps unwittingly, we would expect to see bias of opposite sign across blocks, but Figure 10 shows this did not happen. On the other hand, a combination of (i) an overall response bias toward hedges and (ii) a switching of tendency across blocks, would result in systematically greater bias for the hedge blocks than for the ditch blocks. There is little or no evidence for this asymmetry across blocks in the expert group, but a hint of it for the novices (see Figure 10); we consider the strongest individual cases, that is Novice 6 and Expert 4, below. Where present, these asymmetries could be due to a confounding bias or to genuine sensitivity; our data cannot guide us on the matter. However, since most of the differences are small, it is unlikely that our *d′* analysis for the factor in question (see Figure 11a) is substantially undermined. Nonetheless, there is a possibility that a minor non-stationarity has amplified our estimates of *d′* for the novices for greyscale image information.

In contrast to the concerns above, another possibility is that a tacit expectation of an even distribution of hedge and ditch greyscale images across each block biased participants away from that factor by splitting their responses evenly across the two categories. The substantially non-zero biases in Figure 10 indicate that if this strategy were at play, it was not completely successful, but we cannot rule out an effect, in which case we might have underestimated *d′* for grey-scale image information (see Figure 11a). However, this image factor receives little further attention from us, our emphasis being on the other two factors (disparity and lighting direction) when considering the *d′* results, and these are substantially immune from the nuance of non-stationarity owing to the interleaving of conditions within blocks.

Finally, we return to the two observers in Figure 10 who showed a strong difference in bias across the two block types, beginning with Novice 6. The pattern for this observer is to be expected if there is a tendency to correctly discriminate the grey-level profiles of hedges and ditches, but the diffuse luminance CI for this observer (see Figure 5) does not support this interpretation (i.e., they were not using a luminance-based template), leaving a non-signal related bias across blocks a distinct possibility and raising doubts about our estimate of the greyscale *d′* for this observer (see Figure 11a). Expert 4 is a little different, since the direction of their asymmetry is opposite to that of Novice 6: superimposed on the overall bias to hedges (shared by all participants) they systematically made the wrong response across blocks (in Figure 10, the “hedge” bias is less for the hedge blocks than for the ditch blocks) leading to their negative *d′* in Figure 11a. For this observer, the CIs in Figure 5 are both quite distinct. Furthermore, this observer also has negative *d′* for lighting direction (see Figure 11c) of a similar magnitude. Since it is difficult to attribute this to bias (owing to the signal interleaving), we suspect that this observer used a genuinely inverted detection strategy. We return to Expert 4 in the General Discussion.

#### Sensitivity to disparity profiles

Figure 11b reveals a clear difference across groups for sensitivity to disparity profiles. The average expert sensitivity was *d′* = 0.264 for the three hedge images and *d′* = 0.277 for the three ditch images (not shown). Overall, novices were less sensitive than experts for both hedges (*d′* = 0.046) and ditches (*d′* = 0.051; not shown), and within each ranked pair of observers across groups, the expert always had a greater sensitivity to disparity than did the novice (H1: *t*(10) = 3.51, *p* = 0.003; one-tailed). We also note (i) that the similar accuracies for the hedge and ditch images within group confirms that the stereoscopic profiles of the two image classes were perceived equally well, and (ii) that the generally low performance levels indicate that the disparity noise was an effective mask. (For an unbiased observer, a *d*′ of 0.3 corresponds with 56% correct in a single interval yes/no task.)

**Figure 12.**
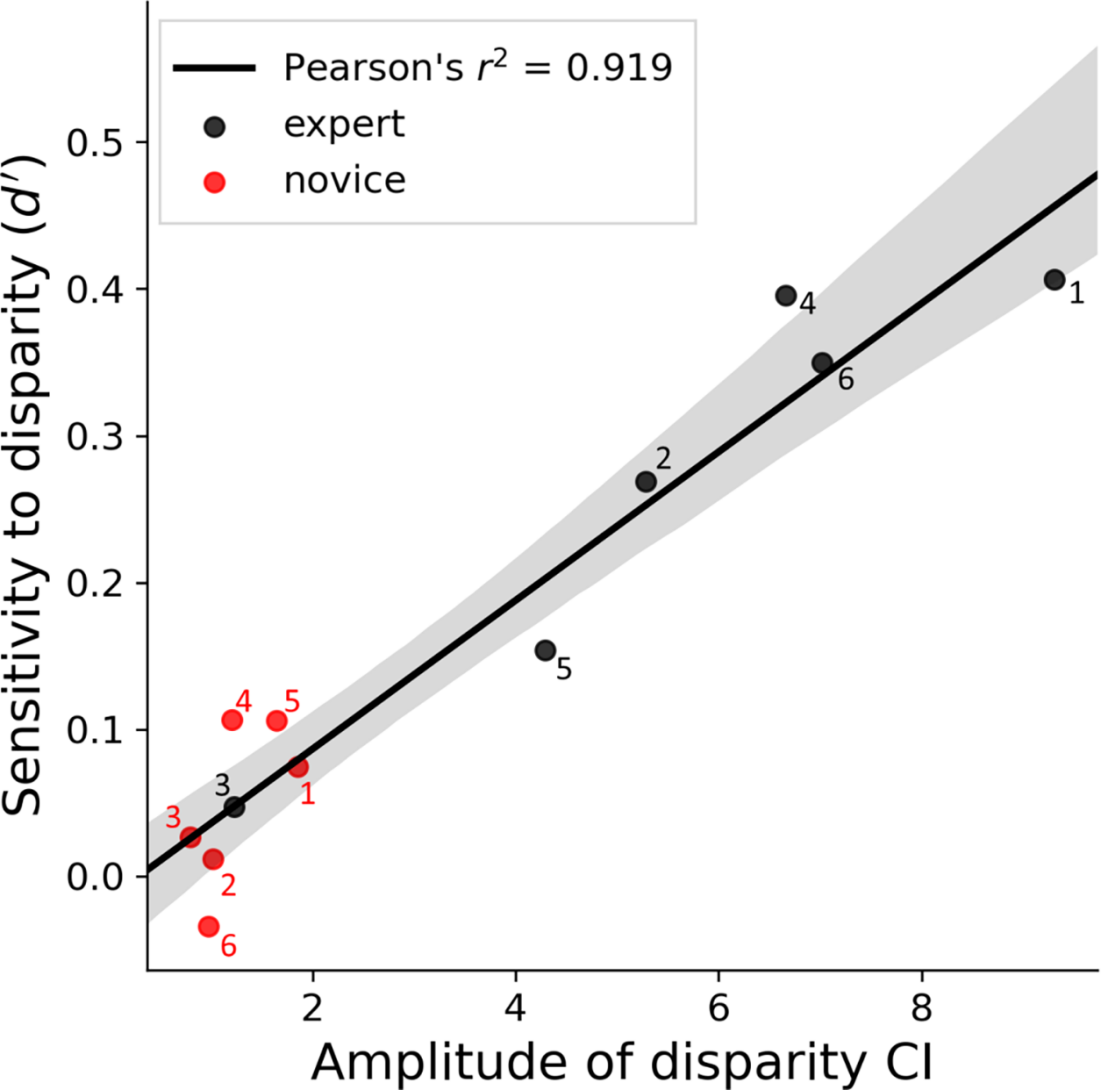
Relation between the amplitude of the disparity classification images (see Figures 5 and 6b) and sensitivity (*d′*) for classifying stimuli as a hedge or a ditch according to whether the landscape feature was presented with crossed or uncrossed disparity (see Figure 11b). Individual participants are numbered (nominally) within the groups.

Figure 12 shows that across observers, *d′* sensitivity for disparity correlated strongly with the amplitude of the disparity CIs (*A* from the Gaussian fits to the individual cross-sections in Figure 6b; Pearson's *r*^2^ = 0.919, *p* < 0.001). This reaffirms our observations that overall, experts had higher scores for both measures compared to novices (H1). This was to be expected because a high amplitude in a CI (in this case, disparity) indicates a high global sensitivity for the relevant parameter which is therefore identified (detected) more reliably. On the other hand, *d′* for disparity did not correlate with TNO thresholds (see Table 1; *r*^2^ = 0.01, *p* = 0.755) even when Expert 5 was removed as a TNO outlier (*r*^2^ = 0.14, *p* = 0.259). This is reminiscent of our earlier (and presumably related) observation that TNO thresholds did not explain group differences in disparity CIs.

#### Sensitivity to lighting from above

Figure 11c shows sensitivities (*d′*) to lighting direction, where values greater than and less than zero indicate perceptual assumptions for lighting from above and below, respectively. This shows that: (1) novices were generally more prone to directional biases (were more distant from zero) than experts, and (2) novices had stronger biases toward lighting from above than did experts, some of whom were biased toward lighting from below (H2: *t*(10) = −1.92, *p* = 0.042; one-tailed). In fact, within each ranked pair of observers across groups, the novice always had a greater sensitivity to lighting from above than did the expert. This supports our hypothesis that expert experience with OS images lit from below the line of sight would diminish the conventional assumption for lighting from above (H2).

Finally, we also note from Figure 11 that across the three different assumptions for ground truth (a, b, and c), the greatest sensitivities were for lighting direction (see Figure 11c), and this was for the novices. We return to this point in the General Discussion.

### Interpreting luminance CIs and individual differences

The differences between groups for the luminance CIs was less than for the disparity CIs (see Figure 5), but several comparisons are worthy of note. First, novices produced larger amplitudes (*A*) than experts (right of Tables 2 and 3; red and black curves in Figures 8b and 8d; see Figure 9). We made no a priori prediction for this result, but it is consistent with the notion the two groups might prioritize information differently. However, we also note that experts had greater spreads (*σ*) in the horizontal direction than did novices (see Table 2). This shows that experts sampled luminance over a greater spatial range of the landscape feature despite giving it lower priority. The group level results from the luminance CIs show that this cue was used by both novices and experts in the task (see Figures 5, 7).

We predicted that patterns in the luminance CIs would relate to lighting direction biases and reveal group differences (H2). The average luminance CIs for both groups (see Figure 8b) show asymmetries, as revealed by the positional offset of the peak (*P*) and relative phase (*φ*) (see Table 3), consistent with lighting from above. These effects were larger for the novices than the experts (see Table 3). For the expert group, the peak was located much more centrally than for the novice group (see Figure 8b), suggesting that the assumed light source was more diffuse for the experts. However, the luminance CIs were less marked overall for this group (see Figures 5, 7), with less asymmetry (smaller relative phase shifts) and (as noted above) lower amplitude (see Table 3). This suggests that the expert group was less prone to lighting assumptions and to lighting and/or luminance cues in general.

**Figure A2.**
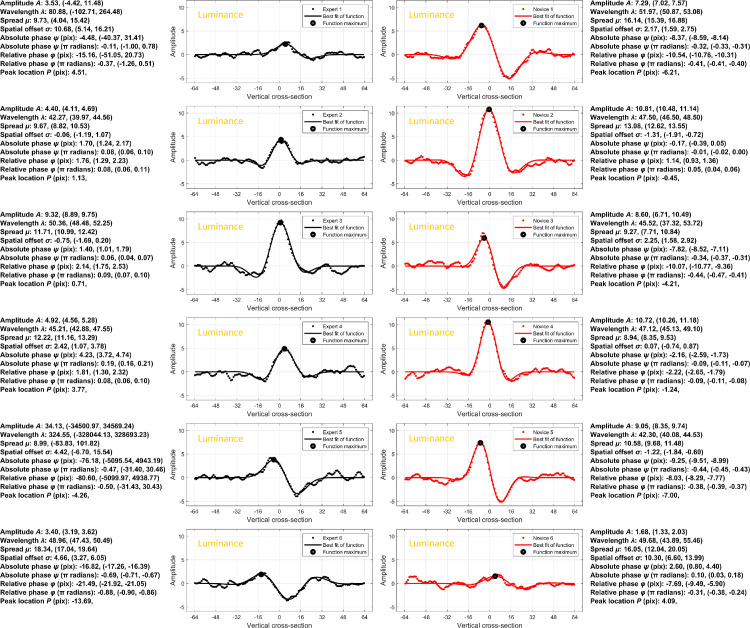
Vertical cross-sections of luminance classification images fitted with a Gabor function for each participant. Details are as for Figure A1.

The analysis above is for group trends but, as Figure 7a shows, there were marked individual differences in amplitudes, lateral peak locations, and phase asymmetries within both the novice and expert groups. This suggests individual differences for assumptions about lighting in terms of both direction (above/below) and source (punctate/diffuse). To examine this, we fitted Gabor functions (Equation 3) to the individual luminance CIs from Figure 7a (see Figure A2 for the fits). In all cases but one, the amplitude was positive and the absolute value of the relative phase shift was less than 0.5*π* radians, consistent with a dark is deep strategy. The exception was Expert 6, for whom the relative phase shift was −0.88*π* radians, placing dark and light pixels more centrally for “hedge” and “ditch” responses, respectively (see Figure 5, bottom left). We have no explanation for this participant's switch in polarity from our expectations, but we note they were one of only two experts who detected the cue for lighting from above (see Figure 11c).

**Figure 13.**
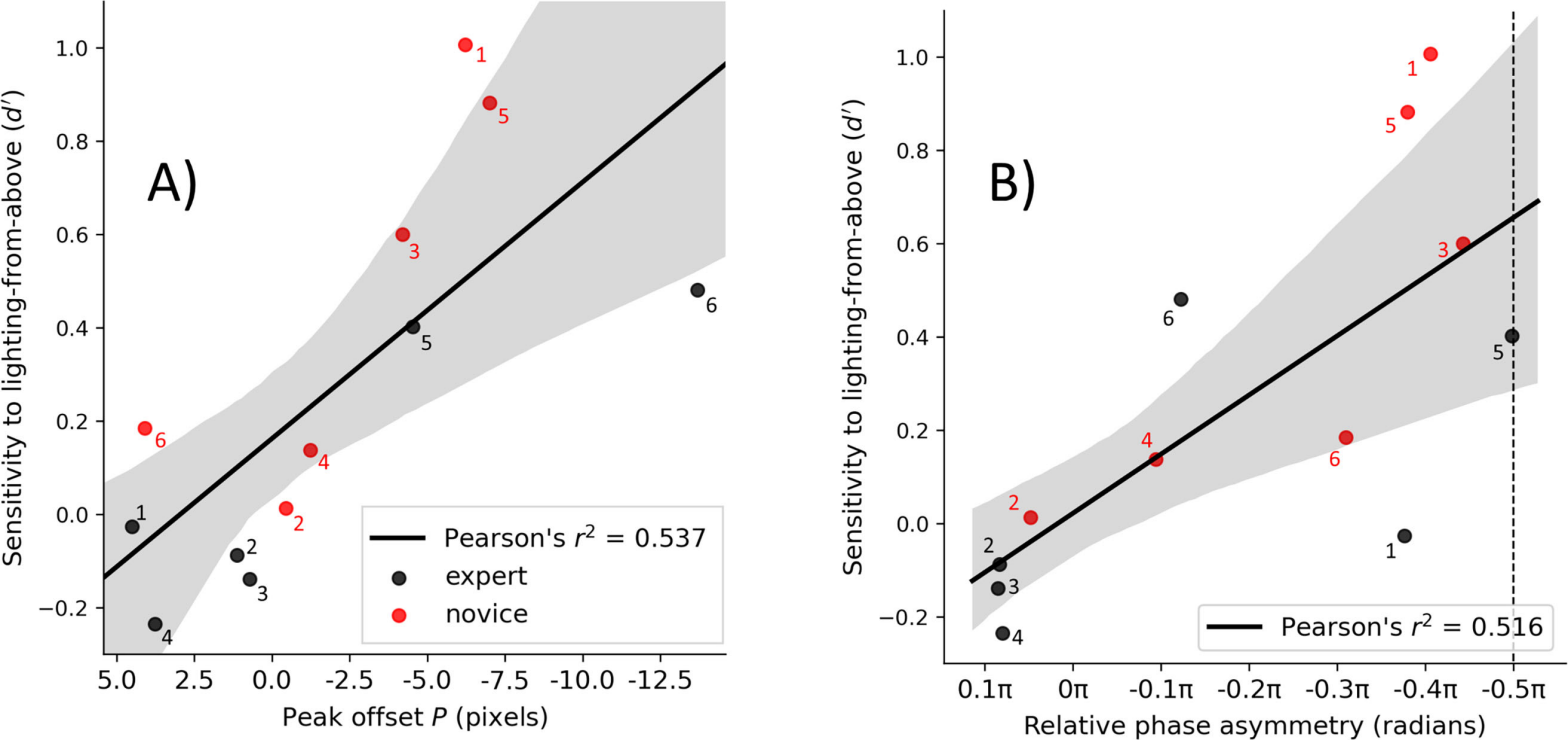
Relationships between sensitivities to lighting from above (see Figure 11c) and peak location offsets (**A**) and relative phase (**B**) in individual luminance classification images. In panel (**B**), a phase of 0π radians indicates perfect cosine phase symmetry, and ±0.5 π radians is maximally asymmetric sine phase (vertical dashed line). Individual participants are numbered (nominally) within the groups. In panel (**B**), Expert 6 was unusual in that |φ| > 0.5 π. Since our aim was for this figure to illustrate asymmetry in the CI, the result for Expert 6 was folded back across −0.5 π. The result is a value of −0.12 π radians under this metric but with opposite sign (light/dark; not depicted in this figure) compared with other participants.

To visualize the individual differences and to show the relationship between the luminance CIs and the categorical results, we plotted the *d′* sensitivity for lighting direction (from Figure 11c) against our two indices of asymmetry: (i) the lateral offset of the function peak in the vertical cross-sections (*P*) and (ii) a metric related to relative phase (*φ*) which tells us about the asymmetry of the shape of the CI. These are shown in Figures 13a and 13b, respectively (see figure caption for details of how the relative phase metric was derived to accommodate Expert 6). In both cases, the correlations were good (Pearson's *r*^2^ = 0.537, *p* = 0.007; Pearson's *r*^2^ = 0.516, *p* = 0.009 in Figures 13a, 13b, respectively). The division across participant groups (different colored symbols in Figure 13) also helps to illustrate the lighting bias hypothesis (H2) where we anticipated that conventional lighting cues would be a more important factor for novices than for experts. These are most marked at the extremes (red symbols, top right; black symbols, bottom left). However, there is marked overlap in the central regions of the plots, showing that the two groups do not delineate as strongly on this measure (see also Figure 5) as they did on disparity (see Figure 12). We highlight some of the details below.

Observers with the strongest perceptual biases for lighting from above (e.g., Novices 1, 3, and 5; see Figure 11c) also had an asymmetric CI with a negative side-lobe “south” of the positive peak (see Figures 5, 7a, 13b). This suggests that a shadow was inferred “south” in hedge features, and/or a highlight “south” in ditch features, consistent with a punctate lighting rule. The opposite inferences of highlights and shadows is seen for observers with a bias for lighting from below (e.g., Experts 2 and 4; see Figures 5, 7a, 13), but less strongly, presumably due to their weaker biases (see Figure 11c). The observers with centralized luminance peaks (e.g., Expert 3 and Novices 2 and 4; see Figures 5, 7a) also showed weaker lighting direction biases (see Figures 11c, 13). This is consistent with a conjunction of (i) diffuse lighting and (ii) “dark is deep” identification rules, where lighter and darker textures prompt “hedge” and “ditch” responses, respectively.

In summary, lighting direction biases are implied by *d*′ sensitivities to lighting from above (see Figure 11c), and peak offsets and asymmetries in the luminance CIs (see Figures 8b, 13). Novices showed a greater tendency for lighting from above, and lighting biases for experts tended to be diminished by comparison or switched to lighting from below. Novices and experts thus differ in their tacit assumptions about lighting and the influences these have on their luminance CIs though overlap between the two groups was marked (see Figure 13).

## General discussion

Using a novel CI technique involving dual forms of external noise applied to 3D images, we investigated two hypotheses (see Introduction) about the influence of expertise in photogrammetry on the perception of 3D relief. We found clear differences in the perceptual templates used by expert and novice observers when interpreting hedge- and ditch-like features in aerial scenes. Our results showed that experts made better use of binocular disparity cues than did novices. Experts also prioritized disparity over luminance, whereas novices prioritized luminance cues over disparity (H1) and had a larger group amplitude in their luminance CI than the experts. Sensitivity to stereoscopic profiles was greater for experts than novices, but this did not relate to stereoacuity. There were individual differences among observers in the interpretation of lighting direction cues, with experts less likely to adopt the conventional prior for lighting from above (H2). We attribute this last result to photogrammetric experience with counter-conventional lighting conditions for the experts.

### The novelty of our CIs and our study

To advance what can be achieved in CI studies, we introduced two novel features to CI stimulus design. First, earlier experiments on disparity used dense (Neri et al., 1999) or sparse (Gosselin et al., 2004) random dot stereograms involving stereo pairs of uniform backgrounds sprinkled with randomly placed dots. In these stimuli, the differences between dot positions across eyes derive from signal and noise (random jitter), and disparity CIs are measured using methods similar to ours. With this approach, the target image is discontinuous. This works well for artificial laboratory-based targets (e.g., a square, a (superstitious) cross), but less so for real photographic imagery. Our use of pixelated images with an algorithm for subpixel disparities overcame this problem, increasing the range of stimuli that can be explored. Second, previous CI studies have not considered luminance and disparity simultaneously (although, see Neri & Levi, 2008 for a study which jointly considered disparity and motion). Since our stereoscopic stimuli were derived from grey-level pixelated images in the first place, it was simple to add luminance-noise as a second independent source of perturbation. With this new approach, we were able to measure pairs of CIs (disparity and luminance) simultaneously for photographs of natural scenes. This was of value because it allowed observer strategy to be investigated across visual cues as well as across groups (novices and experts).

### The shape of the CIs, visual mechanisms, and visual attention

The luminance CIs and the disparity CIs both contained distinct positive (white) and negative (black) regions; typically, a central peak with one or two negative side-lobes. These profiles are reminiscent of the receptive field profiles of neurons in the early visual system which have band-pass tuning characteristics owing to this opponent spatial structure. Such cells have been found for both luminance contrast (Hubel & Wiesel, 1959) and disparity (e.g. Cumming & Parker, 1997) and comparisons have been made between these and CI profiles (e.g., Neri et al., 1999), the implication being that the CIs reveal the structure of the underlying neural mechanism. In fact, the structures of the novice luminance CIs (for example) are not dissimilar to the receptive fields of cortical simple cells, although it might be slightly more spatially extensive for Novice 1 and Novice 5 (see horizontal spreads in Figure 5). Nonetheless, we draw caution. The CI technique involves many trials, and there is no reason to suppose that the same visual neurons are involved in building all parts of both the positive and negative regions of the CI on each trial. For example, if an observer adopts the strategy that hedges (unlike ditches) will produce more crossed disparity than their surrounding environment, then it would be reasonable to treat uncrossed disparity either above or below the target region as evidence for such an arrangement. On this basis, local patchwise analyses of crossed and uncrossed disparities alone might be used to build up the overall picture: the classification image. It is this more conservative interpretation of CIs that we adopt: that the structure of the CI provides information about the observer's overall strategy. Nonetheless, for both luminance and disparity, it is evident that signal contrast is an important component in the overall classification.

A remarkable feature of our disparity CIs is that the midpoint of their vertical cross-sections is very close to zero (i.e., halfway between the top and bottom of the image). This implies that our subjective attempts to locate our hedge- and ditch-like stimuli centrally were successful, since there was no reason to suppose that the disparity CIs would otherwise be asymmetric. From this it follows that a general “dark is deep” strategy with an assumption of diffuse lighting should also produce a similarly placed luminance CI. In fact, we found systematic deviations of the midpoint of the luminance CI for several observers as well as asymmetries in their shape. We discuss the meaning of this in the next section but emphasize here that their inconsistency with the disparity CIs, their variability across observer, and the asymmetries in their shapes, rule out an explanation in terms of systematic deviation of feature placement in the display.

Another notable feature of our CIs was that their horizontal spread (see Table 3) was not as wide as the stimulus feature, indicating that some relevant signal was being ignored. There are several possible reasons for this. First, it might be that the CIs really do reveal details of individual mechanisms, and that the ones in central vision are limited in width. However, psychophysical experiments on contrast detection (Meese, 2010; Meese & Summers, 2012), contrast discrimination (Meese & Summers, 2007), contrast matching (Meese, Baker, & Summers, 2017), contrast CIs (Baker & Meese, 2014), and size perception (Meese & Baker, 2023), all show that for appropriate tasks, the spatial integration of luminance contrast extends well beyond typical receptive field size in the primary visual cortex. Another possibility is that the visual system's general loss of sensitivity and resolution with eccentricity (e.g., Baldwin, Meese, & Baker, 2012) means that more eccentric image regions were of less value to the observer than central ones. One way to address this in the future is to use stimuli that compensate for the eccentric decline (e.g., Baldwin & Meese, 2015). A third possibility is that the constraints relate to the observer's attention window which has been referred to as a spotlight (Posner, 1980) or Gaussian gradient (Downing & Pinker, 1985). Perhaps the high task demands here were such that observers shrunk down the size of their attentional window to remove distraction. For example, Castiello and Umiltà (1990) found that observers can reduce the size of their window of attention to increase efficiency, and that efficiency decreases gradually with distance from the attentional focus. Notably, our experts here prioritized disparity and this is where the highest CI amplitude was found, suggesting that focusing of attention is enhanced by experience. Bosten et al. (2015) also concluded that visual attention was an important factor among unpracticed participants in tests of stereoacuity. Finally, we also note that for our expert group, the luminance CI had a broader horizontal spread than the disparity CI. This is consistent with the idea that the cue of lower priority received less focused attention.

### Novices and experts

**Figure 14.**
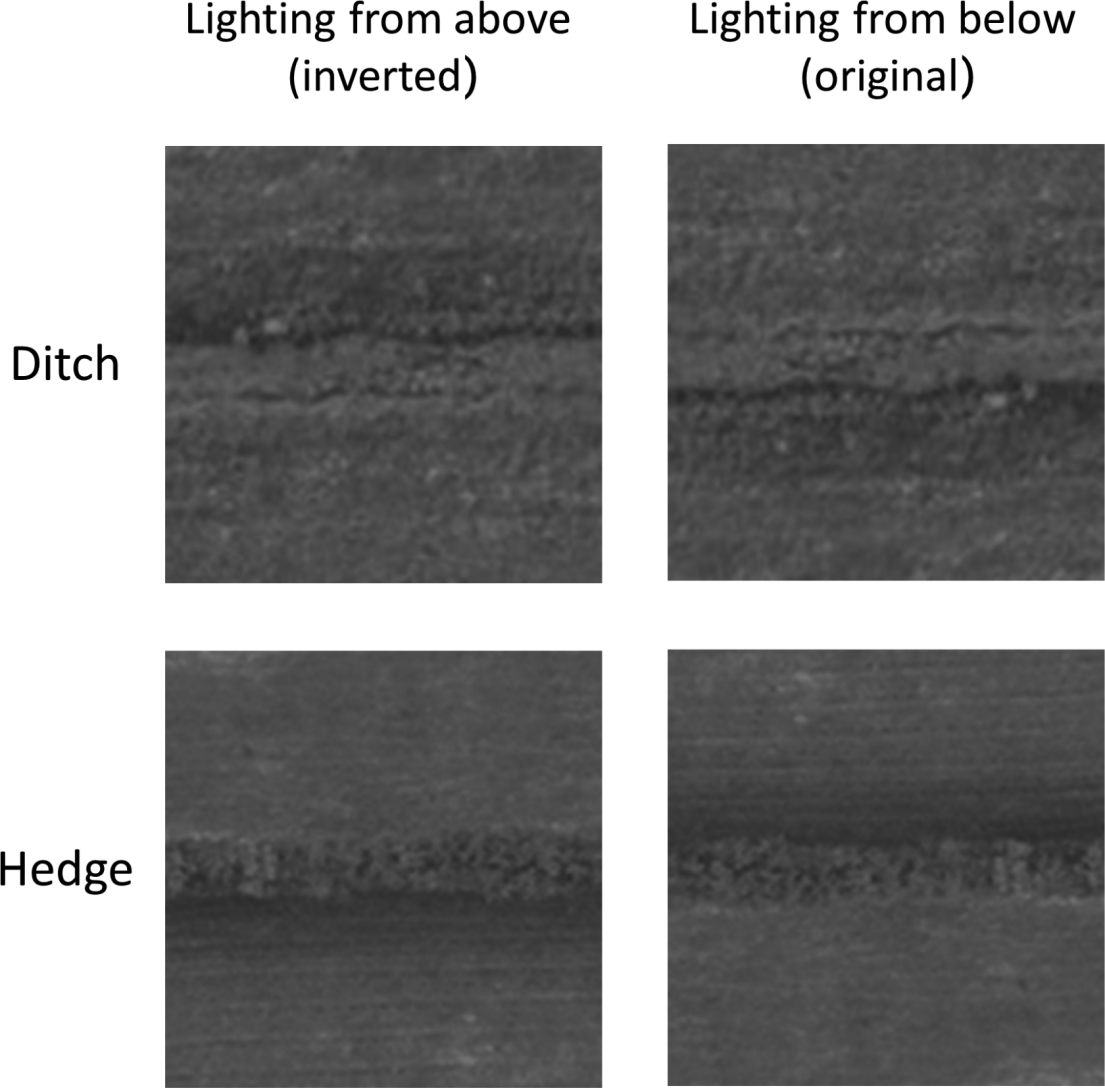
Examples of a ditch (top row) and a hedge (bottom row) taken from Figure 1 (right column) and inverted (left column) to compare the influence of lighting direction on the perception of 3D relief. © *Crown copyright and database rights 2024 OS*, used with permission.

Our OS experts had many hours experience with aerial disparity image pairs. We found that (i) they made better use of disparity cues than novices (as expected) and (ii) they prioritized these over “dark is deep” luminance cues. Less expected was that the novice group would have a greater luminance CI amplitude than the expert group. Furthermore, the novice's *d′* sensitivity for the (implicit) detection of lighting from above was greater for this cue/group combination than any other. This is striking for two reasons. First, from debriefing, it was clear that none of our observers was aware of using the lighting direction cue, although several identified the value of a more general “dark is deep” rule. Second, although lighting direction can be a powerful cue in principle (see Figure 4), we suspect it was rather less important for our clean hedge and ditch stimuli. For example, consider the image content in Figure 14 (try to ignore labels in the first instance) and decide whether each item is a hedge or a ditch. Then, for the top row, decide which is more ditch-like, and for the bottom row, which is more hedge-like. We consider this further below.

Recall that the OS photographs were taken with lighting from the “south”—the bottom of the image. This means that the “inverted” images on the left of Figure 14 are consistent with lighting from above the line of sight, which we refer to as lighting from above. Our impression of these images is that the inversions do not have large perceptual effects. For example, perhaps the hedge on the bottom left has a more robust 3D appearance than the one on the right but the effects are subtle and for us, the one on the right does not flip in 3D in the way it does for the honeycomb (see Figure 4). Nonetheless, this was the tendency in the experiment (averaged across all six image pairs), particularly for novices, suggesting that for our CI task, where images were heavily embedded in noise, visual mechanisms were being tapped at a different level from what is readily seen in casual inspection of noiseless images. This observation might raise a question about the validity of the whole approach: real world decisions are usually based on clear images; heavy levels of noise are rare, so doesn't an experiment like ours tell us more about the perception of noisy images than the clean ones we are interested in? This criticism is readily leveled where the signal-to-noise ratio is sufficiently high for the target image to be readily seen beneath the noise and where judgments are made on some aspect of image content; in those cases, the perceptual decisions are inevitably based on a blend of the signal and noise, and we learn about the perception of noisy images. In the study here, the target image was barely evident, if at all (see Figure 3), as demonstrated by the generally low *d′* sensitivities. With this approach (as with the superstitious method, where the stimulus is not presented), the CI tells us about the perceptual strategies and/or expectations the observer brings to the task, and this involves a characterization of the features being detected. Nonetheless, we cannot rule out the possibility that these processes differ those in the absence of external visual noise. This point is an issue for any psychophysical study where targets are embedded in external noise; it is a difficult one to address and remains a challenge for future research.

Notwithstanding the above, we have learned that the strategies for experts and novices are different. Importantly, by implication, this also tells us what experts are learning from their experience with aerial photography: (1) To prioritize disparity cues over luminance cues when searching for 3D relief. (2) That the conventional prior for lighting from above can be overcome (fully, in 4 out of 6 cases; see Figure 11c)^5^. On the first point, previous studies have found learning effects for luminance CIs in detection of oriented gratings (Dobres & Seitz, 2010), face and texture identification (Gold, Sekuler, & Bennett, 2004) and position discrimination (Kurki & Eckstein, 2014; Li, Levi, & Klein, 2004), but this is the first time that expertise has been demonstrated for disparity CIs. On the second point, this is consistent with other evidence for the shaping of lighting priors by experience. Adams et al. (2004) found a shift in the assumed direction of lighting following haptic training in which the error in the initial lighting assumption was demonstrated implicitly to their participants. However, the authors concluded that the shift in the assumed direction of lighting would most likely revert to convention once their participants were re-emersed in the naturally lit world. We do not know whether the unusual lighting assumptions found here for the experts would transfer to the real world, but it does appear that, for some of them, the training with counter-conventionally lit aerial images has a long-term influence on a perceptual task (detecting signal in noise) that is very different from their training. We are currently exploring this further. Knowledge of expertise in remote sensing surveying at the OS is based in large part on the workplace observation that experience correlates with better performance in domain-specific visual tasks. The underlying processes of learning for aerial images have only been investigated in a small number of studies (Borders, Dennis, Noesen, & Harel, 2020; Lansdale et al. 2010; Lloyd, Hodgson, & Stokes, 2002; Šikl et al., 2019). Šikl et al. (2019) found that remote sensing experts were better at recognizing aerial-view scenes from memory than novices. In addition, Lansdale et al. (2010) found that experts outperformed novices in discounting irrelevant yet salient features. The current study adds to these results by providing exemplar CIs that help to define the experienced surveyors as an expert group.

### Individual differences and the importance of lighting from above

Although the *d′* sensitivities were low, they were not zero. In particular, the lighting direction cue was reliably and unwittingly detected by several observers, feeding into decisions and, possibly, the asymmetry of their CIs. This “effect despite belief” (see Debriefing section) points to a deeply embedded cue within the visual system, evident in both experts and novices. This makes it all the more remarkable that some of our experts have overcome this bias (see previous section). Furthermore, while our analysis showed differences between groups, particularly for the disparity cue (amplitude of the CIs and *d*′ sensitivity), there was heterogeneity within groups for all measures, particularly for lighting from above, pointing to individual differences in the influence this ecological cue imposes on our task. The implications of this are worth considering. A feature of our experimental design was that the sign of disparity and lighting direction were inconsistent in about half the trials (and consistent in the remainder). This means that for observers who detected both cues conventionally (e.g., Experts 5 and 6) these two cues would have been in conflict about 50% of the time, diminishing the performance that would otherwise be achieved. Note that, in general, on removing the conflict trials from the analysis, *d′* equals the sum of those measured when each of the disparity and lighting from above cues were treated as ground truth. For Expert 6, this is quite a benefit, which is to say that in a task where hedge and ditch images are lit from above and have consistent disparity, this observer would benefit from both cue types. Note that this is not specific to observers who show a bias to lighting from above. Expert 4, for example, shows evidence for detecting lighting from below (see Figure 11c). Since the sign of *d′* in Figure 11c depends only on what we deemed to be the correct direction for lighting, it follows that Expert 4 would also benefit from the combined performance across cues (the sum of the absolute values of the *d′* measures) when hedge and ditch images are lit from below. Our point is that only when both (i) there is inconsistency between the observer's lighting prior and the lighting direction in the image and (ii) there is sensitivity to both cue types, that conflict arises. As noted already, the greatest *d′* sensitivities were found for an assumption of lighting from above for the novices. It follows that, in a task such as ours, but where images are presented without conflict, novices would benefit from lighting from above and would benefit further on being trained to use binocular disparity.

### Mechanisms for binocular disparity

We measured stereoacuities using the TNO test and found that these did not explain the difference across groups for disparity CI amplitudes. Expert 5 had the lowest TNO measure and the second lowest CI amplitude among the experts (only Expert 3 was lower). Nonetheless, Expert 5's disparity CI was clearly defined and of greater amplitude than any novice, all of whom had markedly better stereoacuity than Expert 5. Furthermore *d′* sensitivity for disparity did not correlate with TNO. In other words, for our sample of observers, facility in our CI task did not depend on stereoacuity. But perhaps this is not so surprising. Stereoacuity concerns the smallest difference that can be detected across binocular retinal images. This presents a very different task demand from extracting meaningful binocular disparities from a sea of noise. A relevant study here is that of Carrillo, Baldwin, and Hess (2020). They measured sensitivity for depth perception in 53 adults for random dot stereograms in a disparity noise-masking paradigm and found no relation between disparity thresholds and the level of external disparity noise that could be tolerated. Thus, both their study and ours suggest that perception of depth from binocular disparity involves at least two stages: detection of image differences across the eyes (disparities) and the pooling of relevant disparity signal against a background of disparity noise to contribute to the perception of depth.

## Conclusions

Our novel approach to CIs has proved capable in revealing different processing strategies across visual cues and visual expertise. This has practical potential for (i) directing visual training and (ii) investigating the basic perceptual mechanisms of human early vision. We expect that our approach could be readily extended to other domains including: color, motion, and pictorial gradient cues for depth perception.

## Supplementary Material

Supplement 1

## Figures and Tables

**Figure A1.**
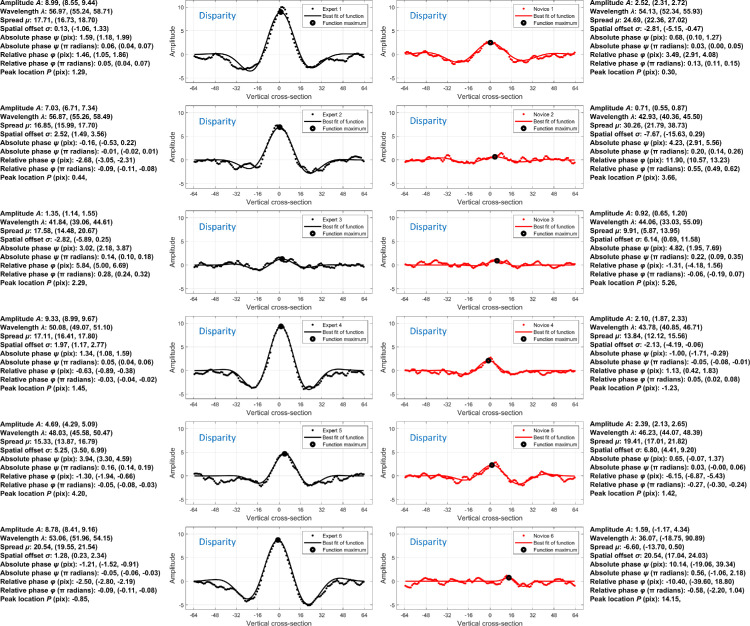
Vertical cross-sections of disparity classification images fitted with a Gabor function (Equation 3) for each participant. Gabor parameter values are listed next to each participant's plot (with 95% confidence intervals shown in parentheses).

## Footnotes

1 In a different landscape classification project involving judgments of craters and mounds on the moon's surface, Bugiolacchi et al. (2016) corrected the orientation of images to ensure the lighting was always from the top-left (Mamassian & Goutcher, 2001; Sun & Perona, 1998). This helps observers to reliably resolve convex/concave ambiguities with the lighting from above prior.

2 The fit to Expert 5’s luminance CI was unusual, bringing our estimate of their amplitude (*A*) into question. The details and our solution are outlined in the Appendix. We also note that when Expert 5 was removed as an anomaly, the conclusion from the ANOVA was unchanged (*F*(1, 4) = 13.16, *p* = 0.022).

3 Some readers might be concerned that our stimulus-response possibilities do not appear to match those in a conventional yes/no detection task. For example, in a yes/no task, a correct rejection involves saying “no” when there was no target stimulus. In our experiment, this is equivalent to saying, “no hedge” (i.e., “ditch”) when there was no hedge—but there was a ditch. In this respect, our experiment is more analogous to phase-reversal discrimination for two-component compound gratings (Lawden, 1983) than it is to simple contrast detection. In the two-alternative forced-choice (2AFC) version of that experiment (Meese, 1995), the observer chooses between compound gratings in peaks-add phase and peaks-subtract phase, and “threshold” is given by the signal level (the contrast of the higher harmonic) at some criterion level of performance (e.g., 75% correct). In other words, the nominal null interval contains a stimulus that is in some sense the opposite of that in the nominal target interval. The 2AFC experiment just described could also be run as a single-interval yes/no experiment (where the target is nominally peaks-add, for example) and with sensitivity derived by calculating *d′*. That arrangement is precisely the one we have here. As an aside, for a range of harmonic relationships, sensitivity for phase-reversal discrimination is twice as good as it is for simple detection of the harmonic against the fundamental. This owes to the presence of an opposite and helpful signal in each interval (Lawden, 1983; Meese, 1995). Although it was of no interest here to compare sensitivity measures for hedge verses ditch compared to hedge or ditch alone, it is reasonable to suppose that a similar relationship to the grating case would apply. In other words, we speculate that our sensitivity estimates here are higher than if session trials were a mixture of hedge (or ditch) stimuli alone and catch trials (i.e., a conventional yes/no target detection paradigm). This nuance neither undermines nor detracts from our analyses.

4 We also speculate that this bias may be due to the speckled nature of the luminance noise mimicking the appearance of hedges more so than ditches. Familiarity with this could also explain the accentuated bias among experts.

5 We have not shown a switch from the conventional lighting bias to an absence of this bias (or its opposite) within observers, so could this difference across groups have come about by chance? An estimate from Schofield et al. (2011) for the proportion of adults that show a conventional bias to lighting direction (i.e., to lighting from above) is seven out of nine participants, which gives a probability of *p* = 0.22 for those who do not. From this, we estimated that the probability of having four or more such observers in a sample of six is *p* = 0.0248, which is statistically unlikely. This is also a conservative estimate. In a study by Pickard-Jones, D'Avossa, and Sapir (2020) using the honeycomb stimulus, all 58 children between the ages of 7 and 11 years showed a bias to lighting from above. In addition, using shaded bubbles, Sun and Perona (1998) found that 12 adults (evenly split for handedness) all had biases to lighting from above.

## Appendix: Fits to vertical cross-sections of CIs for individual participants.

Figure A1 shows best fit Gabor function to the vertical cross sections of CIs for disparity CIs from individual observers. Figure A2 shows similar fits for individual luminance CIs. The fit to Expert 5’s luminance data is unusual. The Matlab optimization routine was drawn towards an unusually high Gabor amplitude (much greater than the amplitude of the data) and a long wavelength. This produced a very shallow sine-wave component to the Gabor function around the zero-crossing that was amplified to reach the data by the high Gaussian amplitude. This unusual nuance had little or no influence on our estimates of relative phase (in π radians) and peak location, which are each reliable indicators of asymmetry but posed a problem for our group statistical analysis of the amplitudes (*A*) from the individual fits. To address this, we tried constraining A, but found the fits were always drawn to the value of our constraint. We then tried constraining wavelength (*l*) but found an interdependence between the value of our constraint and the estimate of amplitude. In a second approach (used in the main body of the report), we calculated the mean ratio between Gabor amplitude and the maximum and minimum values in the data for the other five experts and used this to estimate the Gabor amplitude for Expert 5 from their maximum and minimum values in their data.
