# Supplementary material for: Classification images for aerial images capture visual expertise for binocular disparity and a prior for lighting from above
Source: J Vis. 2024 Apr 12;24(4):11. doi: 10.1167/jov.24.4.11 (PMC11019598; doi:10.1167/jov.24.4.11)
Supplement: Supplement 1 [file jovi-24-4-11_s001.docx]

**Classification images for aerial images capture visual expertise for binocular disparity and a prior for lighting from above**

**Supplementary file**

Emil Skog^1,2^, Timothy S. Meese^2^, Isabel M.J. Sargent^3^, Andrew Ormerod^3^, Andrew J. Schofield^1,2^

^1^School of Psychology,

College of Health and Life Sciences,

Aston University, Birmingham, United Kingdom

^2^Aston Laboratory for Immersive Virtual Environments,

College of Health and Life Sciences,

Aston University, Birmingham, United Kingdom

^3^Ordnance Survey,

Adanac Drive, Southampton SO16 0AS, United Kingdom

**1. Generation of luminance CIs.**

The Luminance CIs presented in the main text were generated by accumulating luminance noise textures before the addition of the signal images and before the application of the disparity noise algorithm. It is common to generate CIs from signal free noise textures in this way but is complicated by the application of disparity noise in our experiment in that the luminance CIs in our main text were not generated from the luminance noise textures presented to each eye. For completeness, Figure S1 shows luminance CIs generated from image pairs displayed nominally on the left and right monitors after applying the disparity noise algorithm but without signal, as before. These CIs were generated via simulations which recreated the experiment, based on the observers’ original responses and the original noise samples presented to each eye subject to our disparity algorithm but without the signal, aerial images. There is no material difference between these alternative CIs and those presented in Fig 5 of the main text.


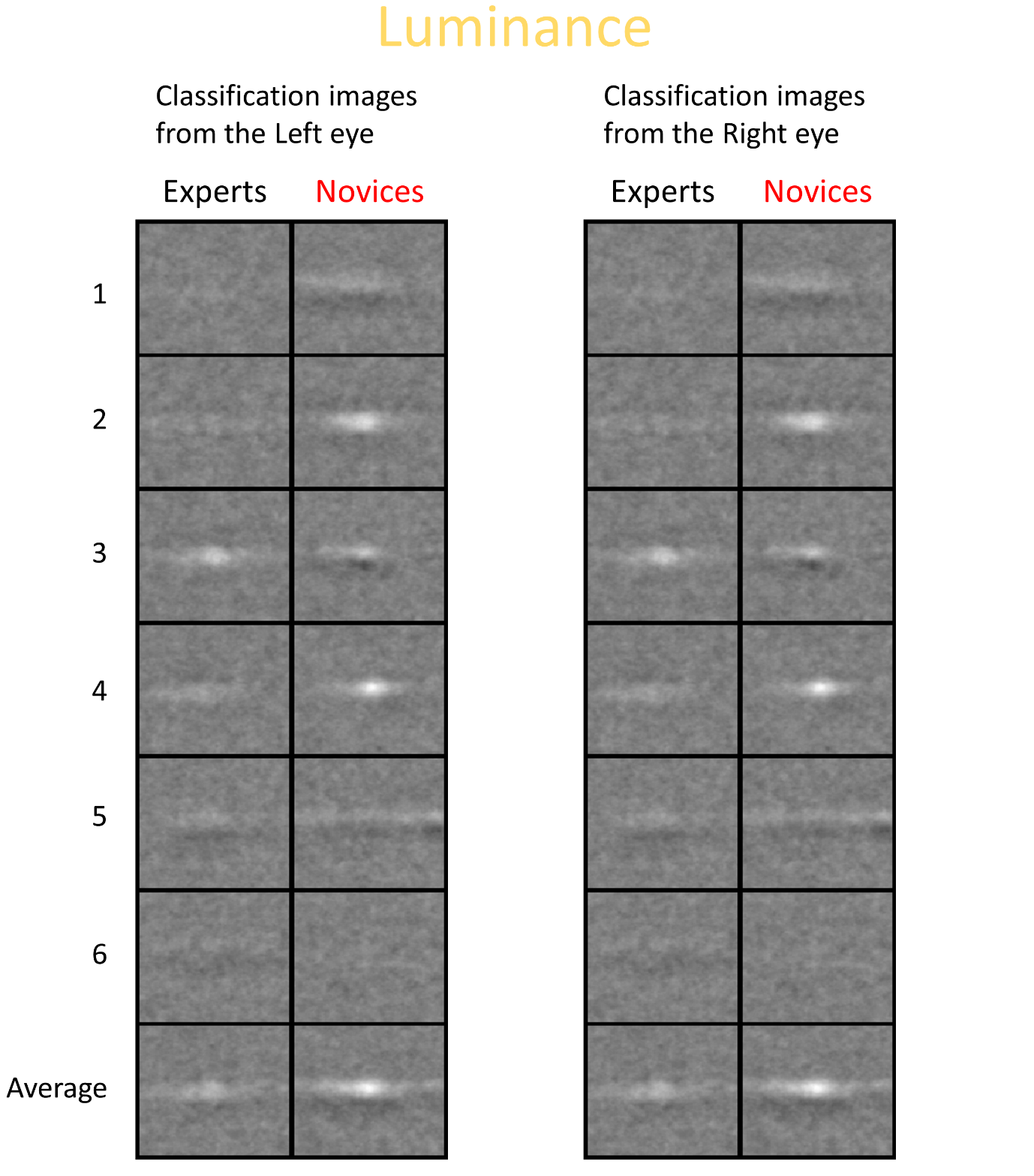


*Supplementary figure S1*: Luminance CIs generated from the noise samples (without the signal images) presented to each eye/monitor after the application of the disparity noise algorithm.

**2. Independence of Disparity and Luminance noise.**

The dual application of luminance and disparity noise raises the possibility that the two signals may become correlated within the image pipeline. This could occur in principle because our algorithm for spatial shifts of sub-pixel disparity alters the luminance values presented to each eye. Generating our Luminance CIs from the original luminance textures sampled before the application of the disparity noise ensured that there could be no direct influence of the latter on the former. Nonetheless it is possible, if unlikely, that our image pipeline introduced some correlation between luminance and disparity in the noise images presented to each eye. To investigate this, we exposed two simulated observers to noise stimuli that were signal free but were otherwise subject to our full image pipeline. The disparity-tuned simulated observer for (Fig S2 left) was programmed to respond ‘hedge’ if the central 20 rows of the disparity noise image contained more crossed than uncrossed disparity and ‘ditch’ otherwise. Its disparity CI reveals this sensitivity while its luminance CI (derived from the average of the two images show to each ‘eye’) shows no structure suggesting that the application of the disparity noise algorithm did not introduce any systematic variations into the luminance images. The luminance-tuned observer was programmed (unlike some of our observers) with a ‘dark-is-deep’ prior and answered ‘hedge’ if the noise in the central 20 rows of the average of the images presented to the two ‘eyes’ had mean luminance above the overall mean, and ‘ditch’ otherwise. Its luminance CI (Fig S2 right) demonstrates this sensitivity, but the disparity CI shows no structure again indicating that the luminance signals as seen by the observers were uncorrelated with the disparity noise.


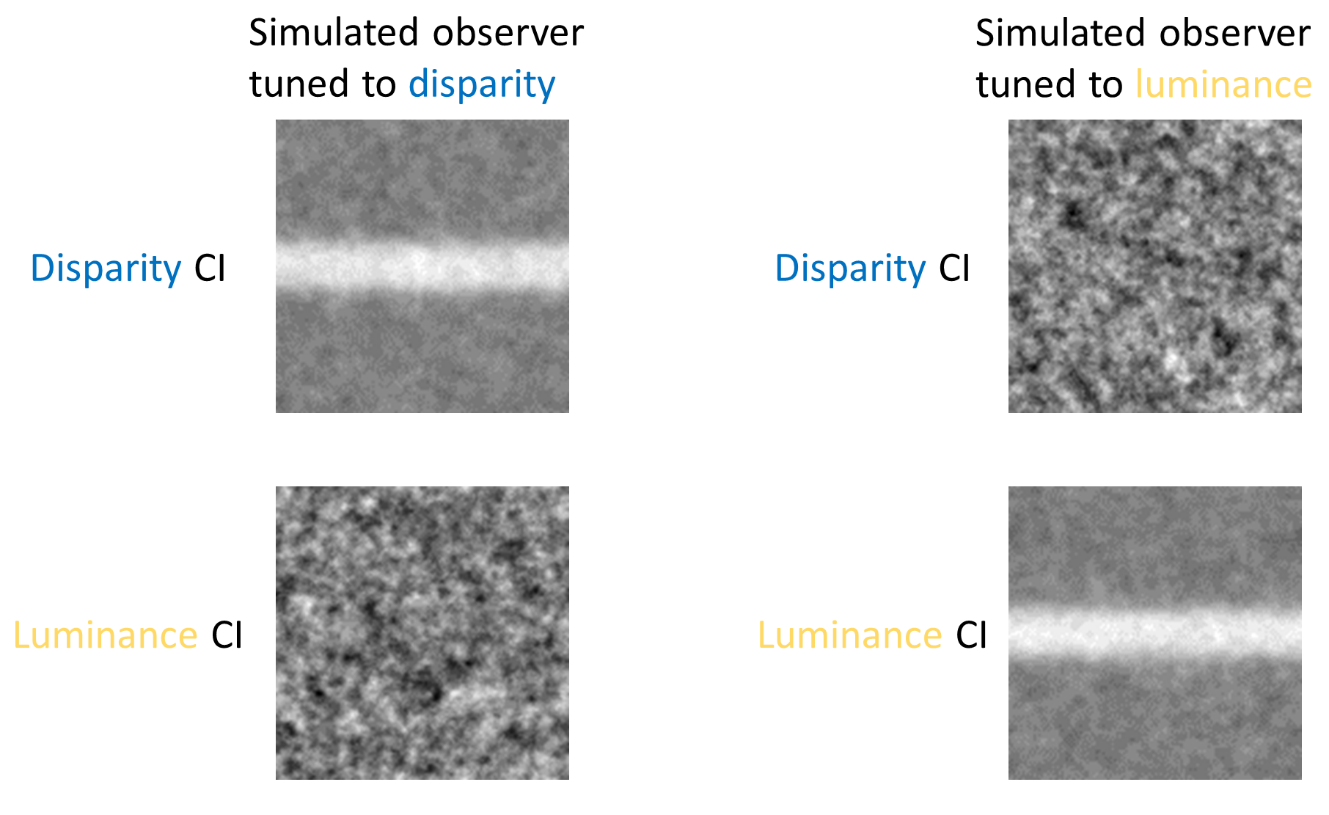


*Supplementary figure S*2: Results of two simulated observers separately tuned to disparity and luminance, after 10,000 trials each.
